# A division-of-labor mode contributes to the cardioprotective potential of mesenchymal stem/stromal cells in heart failure post myocardial infarction

**DOI:** 10.3389/fimmu.2024.1363517

**Published:** 2024-03-18

**Authors:** Xicheng Wang, Chao Yang, Xiaoxue Ma, Xiuhua Li, Yiyao Qi, Zhihui Bai, Ying Xu, Keming Ma, Yi Luo, Jiyang Song, Wenwen Jia, Zhiying He, Zhongmin Liu

**Affiliations:** ^1^ Institute for Regenerative Medicine, Shanghai East Hospital, School of Life Sciences and Technology, School of Medicine, Tongji University, Shanghai, China; ^2^ Shanghai Engineering Research Center of Stem Cells Translational Medicine, Shanghai, China; ^3^ Shanghai Institute of Stem Cell Research and Clinical Translation, Shanghai, China

**Keywords:** CD142, MSCs, scRNA-seq, predictive model, division-of-labor

## Abstract

**Background:**

Treatment of heart failure post myocardial infarction (post-MI HF) with mesenchymal stem/stromal cells (MSCs) holds great promise. Nevertheless, 2-dimensional (2D) GMP-grade MSCs from different labs and donor sources have different therapeutic efficacy and still in a low yield. Therefore, it is crucial to increase the production and find novel ways to assess the therapeutic efficacy of MSCs.

**Materials and methods:**

hUC-MSCs were cultured in 3-dimensional (3D) expansion system for obtaining enough cells for clinical use, named as 3D MSCs. A post-MI HF mouse model was employed to conduct *in vivo* and *in vitro* experiments. Single-cell and bulk RNA-seq analyses were performed on 3D MSCs. A total of 125 combination algorithms were leveraged to screen for core ligand genes. Shinyapp and shinycell workflows were used for deploying web-server.

**Result:**

3D GMP-grade MSCs can significantly and stably reduce the extent of post-MI HF. To understand the stable potential cardioprotective mechanism, scRNA-seq revealed the heterogeneity and division-of-labor mode of 3D MSCs at the cellular level. Specifically, scissor phenotypic analysis identified a reported wound-healing CD142^+^ MSCs subpopulation that is also associated with cardiac protection ability and CD142^-^ MSCs that is in proliferative state, contributing to the cardioprotective function and self-renewal, respectively. Differential expression analysis was conducted on CD142^+^ MSCs and CD142^-^ MSCs and the differentially expressed ligand-related model was achieved by employing 125 combination algorithms. The present study developed a machine learning predictive model based on 13 ligands. Further analysis using CellChat demonstrated that CD142^+^ MSCs have a stronger secretion capacity compared to CD142^-^ MSCs and Flow cytometry sorting of the CD142^+^ MSCs and qRT-PCR validation confirmed the significant upregulation of these 13 ligand factors in CD142^+^ MSCs.

**Conclusion:**

Clinical GMP-grade 3D MSCs could serve as a stable cardioprotective cell product. Using scissor analysis on scRNA-seq data, we have clarified the potential functional and proliferative subpopulation, which cooperatively contributed to self-renewal and functional maintenance for 3D MSCs, named as “division of labor” mode of MSCs. Moreover, a ligand model was robustly developed for predicting the secretory efficacy of MSCs. A user-friendly web-server and a predictive model were constructed and available (https://wangxc.shinyapps.io/3D_MSCs/).

## Introduction

The prevalence of cardiovascular diseases is high, posing a threat to human health and causing a significant economic burden (1). Post-infarction heart failure (post-MI HF) is comprised of acute HF resulting from acute myocardial infarction (AMI) and chronic HF caused by myocardial remodeling after MI. Active treatment is necessary for both acute and chronic HF to optimize the prognosis and minimize the mortality rate of heart failure patients as much as possible. Hence, it is of utmost importance to explore effective approaches that can enhance cardiac function and facilitate the recovery of myocardial ischemic injuries.

Although the functional benefits that have been attained clinically are modest and inconsistent (2), mesenchymal stem cells (MSCs) have gained more and more attention in clinical trials targeting a range of diseases, including heart-related diseases (3–5). Human umbilical cord-derived mesenchymal stem cells (hUC-MSCs) have emerged as ideal cells for the treatment of MI (3, 6). Compared to MSCs sourced from adult tissues, including adipose tissues and bone marrow, hUC-MSCs exhibit longer telomerase and higher activity and relatively lower immunogenicity (7–9). Systemically, MSCs are capable of immunomodulatory and anti-inflammatory effects, reducing inflammation and stabilizing atherosclerotic plaques, thereby decreasing the incidence of cardiovascular events and post-MI HF. In the heart, MSCs can exert their effects through paracrine signaling, ligand-receptor interactions, and immunomodulatory effects, resulting in downregulation of local inflammation, alleviation of tissue inflammatory damage, protection of cardiac myocytes from apoptosis, and improvement of blood flow and energy. Studies have also confirmed the cardioprotective benefits of MSCs in acute MI, whether delivered through immediate intracoronary delivery, intramyocardial or intravenous administration (4, 8, 10–12). However, MSCs in 2-dimensional (2D) culture are in low-yield, and significant differences in therapeutic effects of MSCs were observed from various sources or labs. Therefore, boosting cell production as well as obtaining effective functional estimation tool are of great importance for development of new drug carriers, and clinical stem cell therapies for cardiovascular diseases, especially post-MI HF.

MSC heterogeneity encompasses the diversities observed in therapeutic functions, molecular profiles, differentiation capabilities, cell morphology and more (13). This heterogeneity is not solely attributed to donor origins but arises from distinct subpopulations within a composite cell population (14). In order to understand the heterogeneity of MSCs and the subpopulations within MSCs, there are currently relevant literatures that reveal specific subgroups within umbilical cord MSCs. For example, CD325, a surface marker, contributes to angiogenesis by releasing VEGF, thereby determining the individual differences of hUC-MSC therapeutic effects in a rat model of myocardial infarction (12). Single-cell sequencing and spatial transcriptomics have also revealed that S100A9^+^CD29^+^CD142^+^ MSCs exhibit higher abundance in the fetal segment rather than the maternal segment of umbilical cords, indicating their promising potential as ideal therapeutic agents for wound healing. It demonstrates enhanced tissue regeneration ability in zebrafish wound healing models (14). Single-cell and spatial transcriptomics have also identified a subset of umbilical cord MSCs characterized by limited adipogenic differentiation potential and high expression of BAMBI and MFGE8 (13). Particularly, a study also found that CD168^-^ MSCs have the ability to restore gene expression patterns observed in CD168^+^ MSCs during *in vitro* expansion, suggesting a limited heterogeneity and also the cell plasticity among MSC subpopulations (15). However, it is regrettable that these diverse subpopulations of MSCs may not be stably present in all umbilical cord sources and are not derived under standardized culture conditions. Especially, it is still unclear the relationship and working mechanism of subpopulations in functional MSCs.

Currently, there is no established evaluation model for predicting the therapeutic efficacy of MSCs in treating heart failure, particularly focusing on the secretion capacity of MSCs. In clinical practice, the most important challenge is selecting the best MSCs among thousands of prepared ones for the treatment of post-MI HF. The absence of a comprehensive comprehension concerning the heterogeneity of UC-MSCs and functionally distinct subsets specific to particular diseases still hinders the selection of the most suitable cells required for clinical therapeutic applications. Therefore, it is necessary to identify characteristic molecules that can recognize MSCs with the best *in vivo* efficacy. If there are effective and recommended evaluation parameters/prediction models available, it would enable a more efficient selection of MSCs for HF treatment.

In previous studies, our collaborative team found that intramyocardial injection of 2D hUC-MSCs can survive for >= 28 days in the mouse cardiac tissues and enhance cardiac function. The MSC therapy contributes to angiogenesis in the mouse MI model and alleviates fibrosis and hypertrophy. Intramyocardial administration of MSCs can activate the CCL_5_ pathway to promote the migration of CD4^+^FoxP3^+^ Treg cells to the injured heart (16). However, the treatment efficacy of 2D MSCs was unstable and the cell number of 2D MSCs was still not enough for clinical need. Here, we further expanded the MSCs using three-dimensional (3D) culture to obtained large-scale MSCs. Similarly, we also proved the cardioprotective effects of MSCs cultured in 3D expansion in this study, as the MSCs in 2D expansion did (16). ScRNA-seq technology and machine learning algorithms were leveraged to uncover how 3D MSCs cultured on a large scale can exert therapeutic effects on heart failure, as well as explore the different roles of the subpopulations of MSCs in treating post-MI HF and offer potential estimation tools for estimating secretory and cardioprotective potential of MSCs.

## Materials and methods

### Culture of clinical-use 3D UC-MSCs

The study was conducted and received approval from the Ethics Committee of Shanghai East Hospital of Tongji University. Umbilical cords were collected from full-term infants after obtaining approval from the Ethics Committee and informed consent of the donors. The umbilical cords were transferred to physiological saline and transported at a temperature between 2-8°C. Human umbilical cords were collected, disinfected with a 75% ethanol solution, washed 2-3 times with physiological saline, and cut into small segments of 3-4cm using sterile scissors in physiological saline. After removing the umbilical artery and umbilical vein with forceps, the umbilical cord was cut into small pieces of 1-3mm^3^. The minced cord tissues were evenly spread in a culture flask and incubated in a culture chamber at 37°C and 5% CO_2_ for 2-3 hours. MSCs were collected 5-7 days later. The cells were cultured until passage 3 and then seeded into a 3D low-oxygen bioreactor (BIRUI BIOTECHNOLOGY, Nanjing, China) for expansion, named as 3D MSCs. MSC-microcarriers mixtures were cultured in the bioreactor. The culture environment was maintained at a constant temperature of 37°C and a low-oxygen level of 5%. Cells were collected using a flow cytometer, and surface markers of MSCs were characterized. Flow cytometry analysis confirmed the expression of CD105, CD73 and CD90 on these cells (>95%), as well as the absence of CD11, CD19, CD34, CD31, CD45, and HLA-DR (<2%), consistent with the criteria from the International Society for Cellular Therapy (ISCT) (17).

### Data collection

We collected dataset GSE13491 from the Gene Expression Omnibus database (GEO, https://www.ncbi.nlm.nih.gov/geo/), which includes RNA-seq sequencing data of MSCs for effective and ineffective treatment of post-MI heart failure (12). Additionally, we obtained scRNA-seq data of cardiac progenitor cells previously published by our research group to validate and determine the potential mechanisms of stem cell therapy for post-MI HF through comparing their secretion abilities and screening hub genes and hub cell subpopulations. Other datasets for validation were also collected and showed in **Table 1**.

**Table 1:** All datasets used in the present study.

Source	Platform	Sample	Species	Data type	Team
This work	Illumina novaseq 6000	3D MSCs; 5 samples	Homo sapiens	scRNA-seq	This work
This work	Illumina novaseq 6000	6 normal MSCs; 8 3D MSCs	Homo sapiens	Bulk RNA-seq	This work
GSE13491	GPL570 [HG-U133_Plus_2] Affymetrix Human Genome U133 Plus 2.0 Array	MSCs; two UCB-MSCs (M01 and M02); worst and best efficacy, respectively, in improving post-infarction LV remodeling	Homo sapiens	Bulk RNA-seq	(12)
GSE165811	GPL16791 Illumina HiSeq 2500	MSCs, 9 blank; 9 2IF-stimulated	Homo sapiens	Bulk RNA-seq	(18)
GSE117837	GPL16791 Illumina HiSeq 2500	umbilical cord MSCs; 361 single cells	Homo sapiens	scRNA-seq	(15)
CRA003338	Illumina HiSeq 2000	cardiac progenitors/cardiosphere-derived cells (CDCs), https://bigd.big.ac.cn/gsa/browse/CRA003338	Mus musculus	scRNA-seq	(19)
GSE167219	GPL1261 [Mouse430_2] Affymetrix Mouse Genome 430 2.0 Array	AD-MSCs; 3 samples of CD73+ and 3 samples of CD73- AD-MSCs	Mus musculus	Microarray	(20)
GSE139073	GPL18460 Illumina HiSeq 1500 GPL20301 Illumina HiSeq 4000	Bone marrow MSCs, 33 rest MSCs, 7 stimulated MSCs	Homo sapiens	Bulk RNA-seq	(21)
GSE224190	GPL20301 Illumina HiSeq 4000	hUC-MSCs; 3 untreated and 3 inflammatory cytokines treated (primed)	Homo sapiens	Bulk RNA-seq	(22)
GSE113857	GPL20795 HiSeq X Ten	bone marrow MSCs were transduced with Lenti EF1-PDGFB and Lenti EF1-GFP to obtained PDGF- and GFP-MSCs	Homo sapiens	Bulk RNA-seq	(23)
GSE150008	GPL21103 Illumina HiSeq 4000	Bone marrow MSCs; 3 untreated MSCs; 3 TGF-β1 licensed MSCs	Mus musculus	Bulk RNA-seq	(24)

### Single cell RNA-seq for 3D MSCs

In this study, 3D MSCs were obtained, and single-cell suspensions were collected. Based upon the Singleron Matrix single-cell processing system, single-cell suspensions of 3D MSCs are loaded onto a microwell chip. Subsequently, the captured mRNA is reverse-transcribed and the cDNA was amplified, fragmented and ligated to sequencing adapters. Constructed by the GEXSCOPE Single Cell RNA Library Kit (Singleron), the individual libraries of 3D MSCs were mixed and sequenced by the Illumina platform (novaseq 6000). With the obtained sequencing data, bioinformatics analysis techniques such as t-SNE, PCA, or clustering methods were applied to analyze the differences between individual cells using Seurat package (25), which allowed for a more in-depth exploration of the changes in single-cell biology features, development, and functional variations of 3D MSCs. COSine similarity-based marker Gene identification (COSG) package was utilized to calculate the markers of each clusters (26) and GO, KEGG and Reactome enrichment analyses were done to better understand the heterogeneity of MSCs.

### Bulk RNA-seq for 3D MSCs

RNA is extracted, purified, and enzymatically digested from 2D and 3D MSCs cells to obtain total RNA suitable for RNA sequencing. The RNA is then reverse transcribed into cDNA through the Reverse Transcription process, and the cDNA library is loaded onto the Illumina platform for paired-end sequencing. The raw data (reads) were subjected to preprocessing and quality control using bioinformatics software, including steps such as removing low-quality reads, filtering adapter contamination and polyA tails, to obtain clean reads, which were aligned to the reference genome using tools HISAT2. The alignment results are then used to perform read mapping, and further transcriptome reconstruction and gene expression estimation are carried out using the algorithm feature counts. Total of 14 MSCs samples were sequenced, including 2D MSCs and 3D MSCs.

### Scissor analysis for identifying secretory and proliferative subpopulations of MSCs

Scissor (Single-Cell Identification of Subpopulations with Bulk Sample Phenotype Correlation) is a widely used software package for single-cell transcriptome data analysis, specifically designed for cell subpopulation analysis and screening of single-cell subpopulations closely related to phenotypes (27). In present work, a scRNA-seq dataset of 5 cases of 3D MSCs from our center was employed, along with 12 open-access RNA-seq data of MSCs related to effectively or ineffectively treating heart failure, to explore the phenotypic association between different cell subpopulations and heart failure. Specific procedures included acquiring three data files: the single-cell transcriptomic matrix of 3D MSCs, the bulk transcriptomic matrix, and the metadata file of bulk samples. Subsequently, the Pearson correlation coefficients were computed for each cell-bulk sample pair to construct a correlation matrix. The regression model between the sample phenotype Y and the correlation matrix S was then optimized. The non-zero coefficients β, obtained by solving the aforementioned optimized model, were employed to identify cell clusters related to the treatment of HF. In this case, scissor^+^ represents the selected cells showing positive correlation with the heart failure phenotype, while scissor^-^ indicates negative correlation. In this study, scissor^+^ cells exhibited high expression of CD142, hence we named them CD142^+^ MSCs, which is reported to be capable of wound healing. Parallelly, scissor^-^ cells were also identified as CD142^-^ MSCs with proliferative ability.

### High dimensional weighted gene co-expression network analysis

HdWGCNA is a toolkit based on calculating weighted gene co-expression network, used to investigate the biological characteristics of genes in high-dimensional transcriptomic datasets (28). We preprocessed the raw gene expression data of 3D MSCs using standard methods, including quality control, normalization, and filtering of genes that did not meet the criteria. Furthermore, we utilized the hdWGCNA package (version: 0.1.1.9010) to build a weighted gene co-expression network: in this study, we employed the default parameter settings to build the gene co-expression network and performed weighted processing for each gene, taking into account their different expression patterns and biological significance. The soft power threshold was set as 4. The gene network was partitioned into 7 different modules, which are subnetworks composed of genes with similar expression patterns. Biological annotations were performed for genes within each module, thus inferring the potential biological processes and pathways associated with these modules. Finally, various visualization tools provided by hdWGCNA were utilized to visually display the gene co-expression network and the biological annotation information of modules, providing deeper perspectives of the heterogeneous biological mechanisms of MSCs.

### Pseudotime analysis

Monocle2 package (version: 2.22.0) provides powerful non-linear dimensionality reduction techniques and cell trajectory analysis tools. Based on Monocle2, we constructed developmental trajectories of various subpopulations within 3D MSCs, including CD142^+^ and CD142^-^ MSCs. “Ordercell” function was used to order 3D MSCs in pseudo-time. Furthermore, we analyzed distinct MSCs subpopulations, their associated molecules, functions, and distribution along each trajectory branch using plot_cell_trajectory function. Moreover, we conducted analysis on ligand genes of 3D MSCs, presented them in pseudo-temporal heatmaps, and explored their interrelationships and mechanisms of action (29).

### Cell-cell communication analysis

In this study, we employed algorithms from the CellChat package (Version: 1.0.0) to classify cells and link them to their respective clusters, creating a cell-cell interaction network for 3D MSCs. Additionally, we augmented the cell-cell interaction network by including important cell types and gene information to observe the interaction features among different units. CellChat also contains a large number of ligand-receptor pairs. We filtered the genes that were significantly upregulated in CD142^+^ MSCs to retain only those encoding hypothetical secreted proteins (ligands) (30).

### Spatial construction of CD142^+^ and CD142^-^ MSCs

CSOmap (Cellular Spatial Organization mapper) in MATLAB is a scRNA-seq data analysis method that utilizes ligand-receptor interactions between cells to predict cellular spatial relationships (31). The MSCs sequenced in this study were cultured using a 3D MSCs culture system, thus exhibiting spatial heterogeneity. We used the CSOmap algorithm to reconstruct the potential spatial re7lationships of MSCs during 3D culture, thereby revealing the spatial structural differences between scissor^+^ and scissor^-^ MSCs. MATLAB (version: R2021b) was used and further visualization was finished using R (version: 4.1.1). All parameters were set as default. The distance to center was calculated using the following formula:


$$Distance\;to\;center=\;\sqrt[{2}]{x^{2}+y^{2}+z^{2}}$$

### Post-MI HF mouse model and MSC therapy

All experiments were approved by the Institutional Animal Care and Use Committee of Tongji University, Shanghai, China. The post-MI HF model in C57BL/6J mice was established by the permanent ligation of left anterior descending coronary artery (LAD). Briefly, each mouse was anesthetized with 1-2% isoflurane, intubated and mechanically ventilated to maintain consistent respiratory rate with the ventilator. Successful intubation was confirmed by observing synchronized chest movements. Each mouse was placed on a heating pad to maintain body temperature and a thoracotomy was conducted in the third intercostal space above the left chest. The LAD was ligated using 6-0 silk suture. Ischemia and blanching in the region between the ligation site and the apex of the heart indicated the successful LAD ligation. For the experimental group, following previously described protocols (16), 3D MSCs under hypoxic conditions were injected at three points into the areas adjacent to the infarcted tissue, approximately 10µl per point, resulting in a total injection of 3 × 10^5^ cells per mouse myocardium (30µl in total). Echocardiography was performed on days 7 and 28 after cell therapy to evaluate cardiac function in mice. Mice were euthanized at different time points for subsequent experiments. Cardiac issue evaluation, as well as RNA and protein extraction, was performed on harvested samples from mice at day 28 post-treatment. There were at least 3 mice in each group.

### Echocardiography for post-MI HF mice with/without MSC therapy

Mice were anesthetized using 2% isoflurane inhalation. Equipped with a 30-MHz linear transducer (FUJIFILM Visual-Sonics, Inc.), a Vevo 2100 high-resolution imaging system was utilized to assess the whole process, following previously described protocols (16). Using M-mode images, cardiac function of the sham and treated groups was evaluated through long-axis scans. Key parameters were calculated and collected both on day 7 and 28 after establishing the post-MI HF model, containing LVFS (left ventricular fractional shortening), LVEF (left ventricular ejection fraction), LVEDV/LVESV (left ventricular end diastolic/systolic volume) and LVEDD/LVESD (left ventricular end diastolic/systolic diameter).

### Hematoxylin & eosin staining

The mice were euthanized and intact heart tissues were extracted. Tissues were immersed in 10% formalin and placed on a shaker for 24 hours to undergo fixation. After fixation, the tissues were dehydrated, embedded, and finally prepared into sections, which underwent heating, deparaffinization and rehydration processes, followed by three washes with PBST (phosphate-buffered saline with Tween-20 detergent), with each wash lasting for 5 minutes. After washing, the sections were stained with hematoxylin, immersing them in a hematoxylin staining solution typically for 5 minutes. Then, the sections counterstained by rinsing them repeatedly in running tap water for 5 to 10 minutes. Afterwards, the samples were dehydrated with sequential immersion in 50%, 70%, 80%, and 95% concentrations of ethanol, with each concentration lasting for 2 minutes, respectively. The sections were stained with eosin by gently applying eosin staining solution for 1 minute. After completing the H&E staining process, the obtained results were documented by photography.

### Immunofluorescence staining

Firstly, the sections were deparaffinized and dehydrated using xylene and were placed in running water for 10 minutes to ensure thorough removal of xylene. 1X repair solution was applied to the sections, which were then heated in a pressure cooker at 121°C for 2-3 minutes. Subsequently, a 3% hydrogen peroxide solution was incubated with the sections in a light-protected 37°C incubator for 20 minutes to eliminate endogenous peroxidase activity. Blocking solution (composed of 0.2% PBSTriton X-100, 1% bovine serum albumin and 10% normal donkey serum) was added to the samples, and they were incubated at room temperature for 45-60 minutes to reduce nonspecific binding. Additionally, the samples were incubated overnight at 4°C for antibody binding by adding a CD31 antibody (diluted to 1:200, Cell Signaling Technology, Cat.77699S). Finally, DAPI staining solution (Solarbio, C0065) was added for nuclear staining, and the results were observed using a fluorescence microscope.

### Masson’s trichrome staining

The sections were immersed in a staining solution and incubated overnight at 37°C. Next, the sections were stained by adding a solution of aniline blue for 2-3 minutes. Subsequently, Mayer’s hematoxylin solution was added to the sections for a staining duration of 2-3 minutes. Then, the sections were briefly treated with an acid differentiation solution, followed by rinsing with tap water for 10 minutes. Next, a solution of Ponceau acid fuchsin was added to the sections for a staining duration of 10 minutes. The sections were treated with a solution of phosphomolybdic acid for 10 minutes. The remaining dye solution was discarded, and the sections were directly stained with aniline blue for 5 minutes without rinsing. An acetic acid working solution was prepared by mixing acetic acid and water in a 1:2 ratio, and the sections were rinsed in this solution for 2 minutes. Finally, the sections were cleared with xylene for 1-2 minutes each time. Ultimately, the sections were mounted using neutral resin. The main reagents used in the above steps were from the Modified Masson’s Trichrome Staining Kit (Solarbio, G1346).

### Flow cytometry analysis

MSCs were trypsinized using TrypLE Express (Thermo Fisher Scientific, USA). After digestion, the MSCs were washed three times with PBS and then resuspended. They were then stained with fluorescent antibodies including CD90 and CD105 (BD Biosciences, 1.25μL/test), as well as CD73, CD11b, CD19, CD31, CD34, CD45, and HLA-DR (BD Biosciences, 5μL/test). The staining was performed at 4°C in the dark for 30 minutes, followed by termination with PBS. The MSC samples were centrifuged at 2000 rpm for 6 minutes, washed twice, and then resuspended. Subsequently, the MSCs were classified using a flow cytometer (BD Stemflow, 562245) to detect specific surface markers. FlowJo software (Tree Star, Ashland, OR) was utilized for result analysis. CD90, CD73, and CD105 were positive while CD19, CD11b, CD31, CD34, HLA-DR and CD45 were negative for our cultured MSCs, consistent with the criteria set by the International Society for Cellular Therapy (ISCT) (17).

### Flow cytometry sorting of CD142^+^ MSCs

To isolate the CD142^+^ MSCs, fluorescent antibodies targeting the positive selection marker CD142 (BD Biosciences, 5μL/test) were used for staining at 4°C for 15 minutes. After centrifugation at 300×g for 5 minutes, the pellet was washed with PBS and the cells were resuspended in PBS to a cell concentration of 1×10^6^ cells/mL. The CD142^+^ MSCs were sorted and analyzed using MoFloAstrios EQ.

### Quantitative real-time PCR

Following the manufacturer’s instructions, the total RNA was extracted using TRIzol regent (Thermo Fisher, 15596018) and the RNA concentration was measured using NanoDrop2000 (Thermo Fisher Scientific). Subsequently, utilizing the PrimerScript RT kit (Takara, RR0036A), cDNA was synthesized. Then, qRT-PCR was employed to assess the mRNA levels with the aid of a SYBR Green Master Mix kit (Thermo Fisher, 4385617). The mRNA expression was normalized to GAPDH and the 2^(-ΔΔCT) method was utilized. The specific gene primers used in the present study were listed in **Supplementary Table S3**.

### Gene set enrichment analysis

To make a better way for identifying key pathways for understanding the roles of CD142, we also adopted the notion of “metacell” (32, 33) in this work, which could better remove/reduce data noise caused by single cell RNA-sequencing technology. Metacell numbers of background cells, scissor^-^ cells and scissor^+^ cells were 1709, 141 and 201, respectively. To better understand the pivotal role of CD142, we also conducted the GSEA pipeline, which could reveal the activations of pathways (34). The clusterProfiler package (35) and fgsea package (36) were leveraged to visualize the significantly activated pathways. To better understand the 13 ligands of ligand model, ssGSEA algorithm in gene set variation analysis (GSVA) package was also leveraged (37).

### Combined algorithms for screening optimal algorithms for constructing ligand-based predictive model

125 combined algorithms were performed successfully and AUC was considered as the parameter for estimating the model performance. These algorithms consist of Lasso, Elastic network (Enet), Ridge, partial least squares linear and generalized linear regression (plsRglm), stepwise glm (Stepglm), glmBoost, support vector machine (SVM), linear discriminant analysis (LDA), RandomForest, Naive_Bayes, adaBoost, generalized boosted regression modelling (GBM), classTree and xgboost algorithms.

### Stratification card for MSC secretory potential assessment

To improve the convenience and flexibility of this ligand-based machine learning model in clinical applications, we employed the Kolmogorov-Smirnov (KS) curve to identify the optimal threshold for MSC secretory potential assessment card. The MSC secretory potential assessment card was mainly composed of five grades of secretory power: “Very Low”, “Low”, “Normal”, “High” and “Very High”. The larger the KS value, the greater the discriminative power of the corresponding threshold in the model. And after validation using external cohorts, we found that the secretory power also indicated the cardioprotective potential.

### Website development

To provide readers/researchers with a more convenient understanding of our work and a more comprehensive grasp of the characteristics of 3D MSCs, we utilized shinycell package (38) for web development and published it on shinyapp (https://wangxc.shinyapps.io/3D_MSCs/). Moreover, we have uploaded the MSC secretory potential assessment card to the server we developed, facilitating researchers to utilize our assessment tool and enabling faster evaluation of the cardiac protective capabilities of MSCs.

### Statistical analysis

The data is shown as mean ± SEM (standard error of the mean). And the “p-value<0.05” is considered statistically significant.

## Result

### Framework of the present study

**Figure 1:**
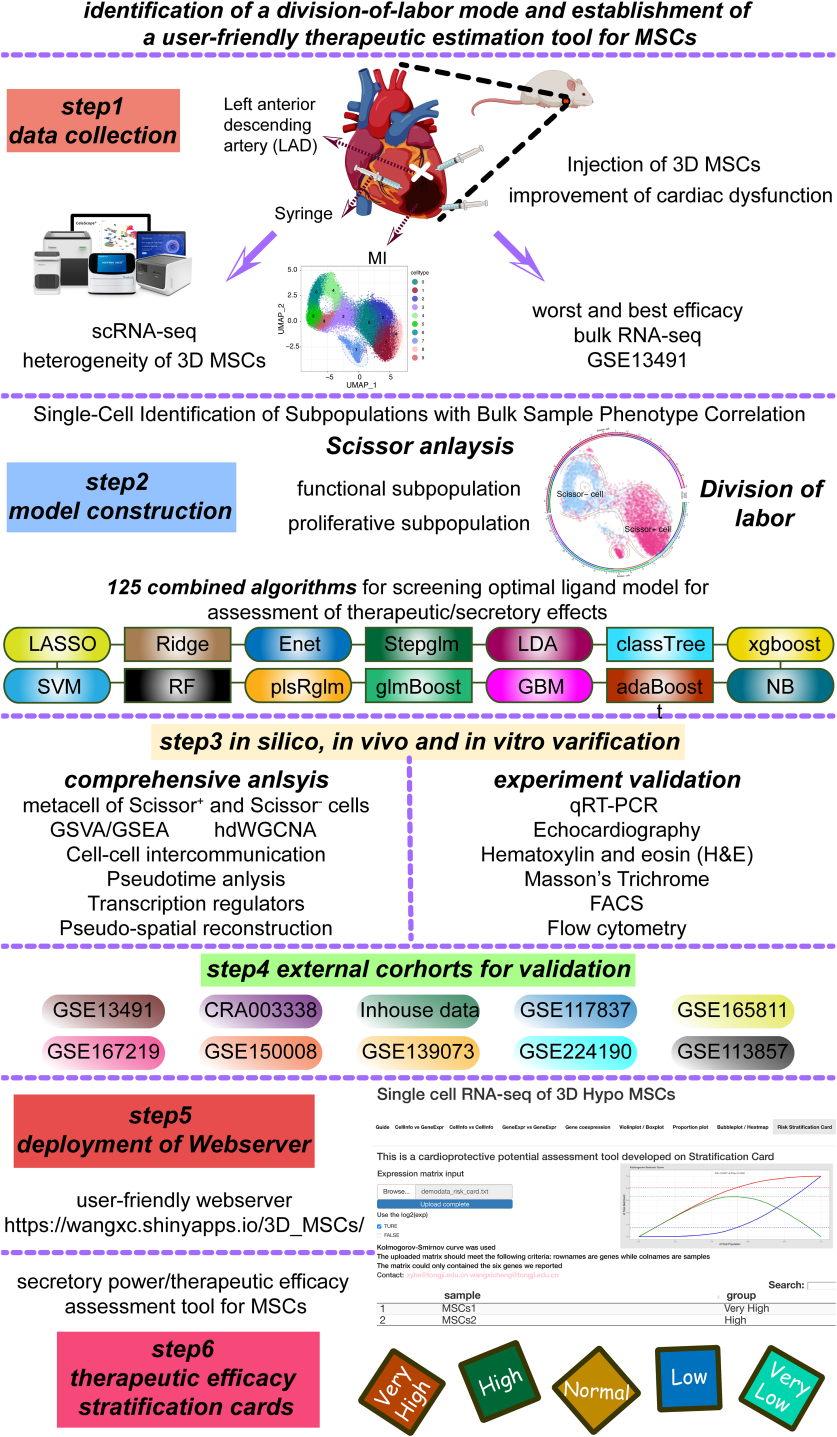
Detailed framework of the present study. Enet, elastic network; Stepglm, stepwise glm; SVM, support vector machine; LDA, linear discriminant analysis; plsRglm, partial least squares linear and generalized linear regression; GBM, generalized boosted regression modelling; NB, Naive_Bayes.

In the previous study, our collaborative team have confirmed the cardioprotective potential of hUC-MSCs, which were cultured in common 2D expansion medium and were able to express CCL_5_ and then recruit CD4^+^ T cells and CD4^+^FoxP3^+^ Tregs for exerting cardioprotection potential (16). However, the clinical usage of MSCs was large while 2D expansion method had a limit in cell yield. And the therapeutic efficacy of 2D MSCs was heterogeneous and unstable. Hence, we tried to expand hUC-MSCs in 3D expansion system, and the expanded MSCs were named as “3D MSCs” here. Here, we would try to estimate the cardioprotective potential of the clinical GMP-grade 3D MSCs and also construct robust estimation tool for predicting the secretory power of MSCs using scRNA-seq and machine learning algorithms. The detailed workflow was showed in **Figure 1**.

### Cardiac function was positively improved by the intramyocardial administration of 3D MSCs in post-MI HF mouse model

**Figure 2:**
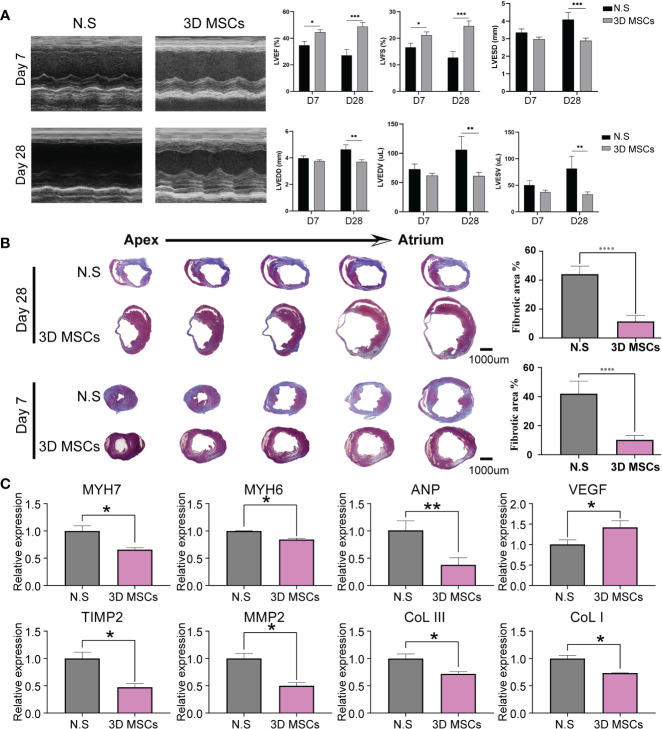
3D MSCs exerted cardioprotective role in post-MI HF mouse model. **(A)** Representative echocardiographic images (M-mode) in two groups on day 7 and 28 post MI surgery. Statistical results of cardiac function on day 7 and 28 between N.S and 3D MSC groups. *p< 0.05; **p < 0.01; ***p < 0.001; ****p < 0.0001. LVEF: left ventricular ejection fraction; LVFS: left ventricular fractional shortening; LVEDD/LVESD: left ventricular end diastolic/systolic diameter; LVEDV/LVESV: left ventricular end diastolic/systolic volume. **(B)** Representative Masson’s Trichrome-stained histological sections of cardiac tissues. Scale bar = 1000 μm. **(C)** mRNA levels of fibrotic and hyper-trophy markers in infarcted hearts. N.S indicates normal saline.

To assess the cardioprotection function of 3D MSCs, by permanently ligating left anterior descending coronary artery (LAD), we constructed the post-MI HF mouse model (**Figure 1**). As displayed in **Figure 2A**, intramyocardial injection of 3D MSCs had a pronounced positive effect on LVFS and LVEF at both day 7 and day 28. Moreover, it led to a significant decrease in LVEDV/LVESV and LVEDD/LVESD on day 28 in the post-MI HF group. Moreover, 3D MSCs treatment ameliorated the average fibrotic area compared to control group on day 28 (**Figure 2B**). Then, we also detected the expression levels of hyper-trophy markers (ANP, MYH7 and MYH6) and fibrosis markers (collagen III, collagen I, MMP2 and TIMP2) (**Figure 2C**). All expression levels of these markers were notably reduced after 3D MSCs therapy, implying that 3D MSCs administration alleviated cardiac hypertrophy and fibrosis on post-MI HF.

### Single cell RNA-seq analysis indicated the heterogeneity of 3D MSCs

**Figure 3:**
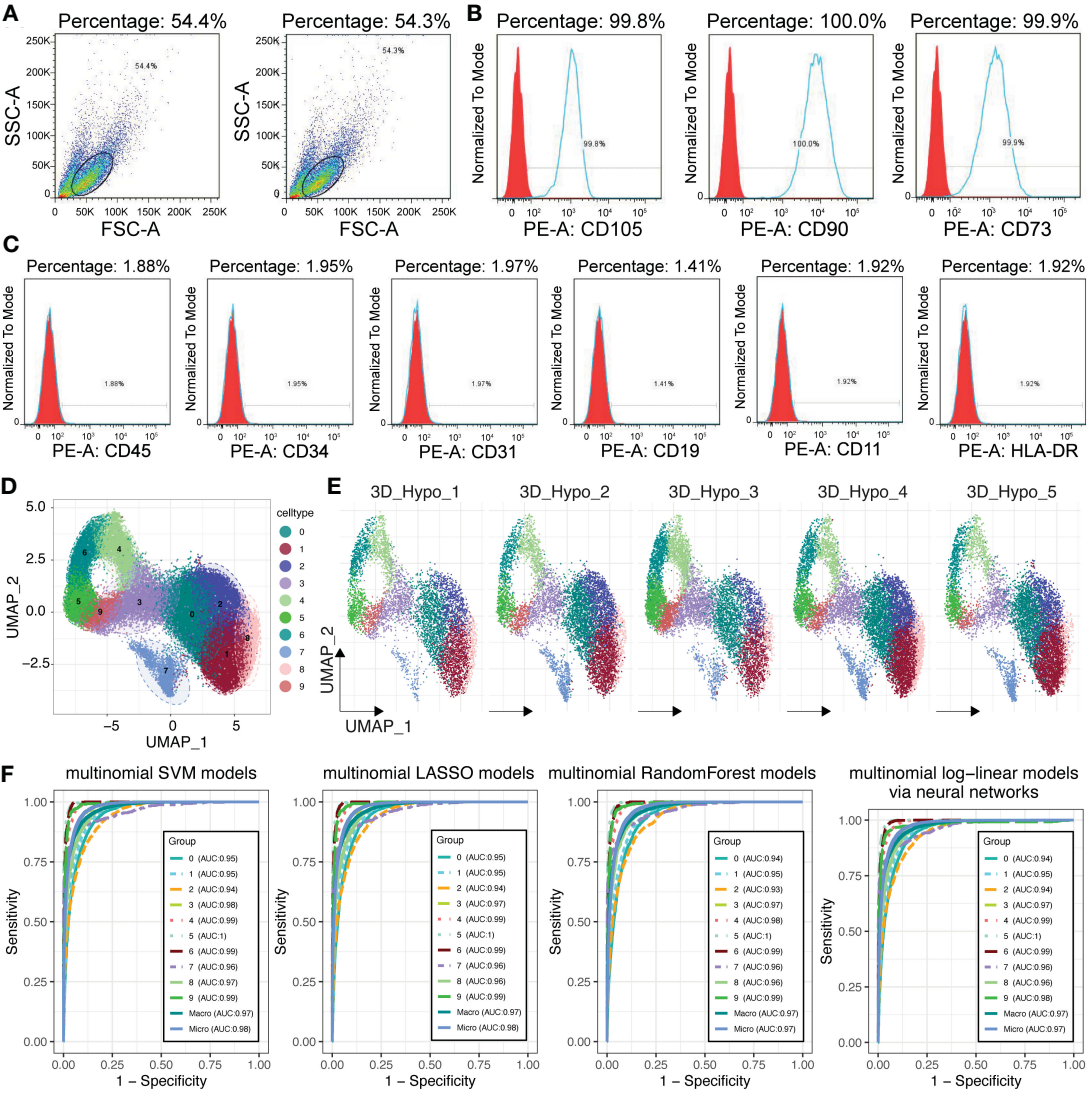
Single cell RNA sequencing for 3D MSCs. **(A)** Flow sorting of MSCs. **(B)** Percentage of CD105^+^, CD90^+^ and CD73^+^ MSCs. **(C)** Positive percentage of CD45, CD34, CD31, CD19, CD11 and HLA-DR. Most of these CD markers for other cell types were negative expression for 3D MSCs. **(D)** Single cell RNA sequencing identified 10 subpopulations for 3D MSCs. Harmony function was used to remove batch effect. **(E)** Subpopulation distribution in each sample of 3D MSCs, showed by UMAP plot. **(F)** Machine learning algorithms for validating the distinction of the 10 subpopulations of 3D MSCs, including SVM, LASSO, Random Forest and multinomial log-linear models based on neural networks. All the AUC values of each subpopulation were showed.

To better study the heterogeneity of MSCs, we performed scRNA-seq in the clinical-use 3D MSCs, which have met the criteria of MSCs built by the International Society for Cellular Therapy (ISCT) (17), comprising the expression of CD105, CD90 and CD73 (>95%) and no presentation of CD45, CD34, CD19, CD31, CD11 and HLA-DR (**Figures 3A–C**). After quality control, we obtained 41181 single cells from 3D MSCs. Using seurat package (25) and clustering analysis, we have got 10 clusters of MSCs (**Figures 3D, E**). To confirm the heterogeneity, we adopted machine learning algorithms for validation. Of interest, several algorithms, including SVM, random forest, lasso and multinominal log-linear model via neural networks, have proved the difference among these clusters (**Figure 3F**). Subsequently, we used COSG algorithm to calculate the cluster marker genes and identify the potential function of these 10 clusters (**Supplementary Figures S1**, **S2**). As showed, C4, C5, C6 and C9 were enriched in “Cell Cycle”. C0, C1, C2, C7 and C8 were mainly enriched in “wound healing”, “growth factor binding” and so on. The detailed enriched terms of each cluster were showed in **Supplementary Figures S1**, **S2** using GO, KEGG and Reactome database. Thus, MSCs have heterogeneity and thereafter it would be crucial to identifying what was the potential working mode for exerting cardioprotection effect of MSCs.

### Scissor analysis identified CD142^+^ MSCs as bio-functional MSCs while CD142^-^ MSCs as proliferative MSCs

**Figure 4:**
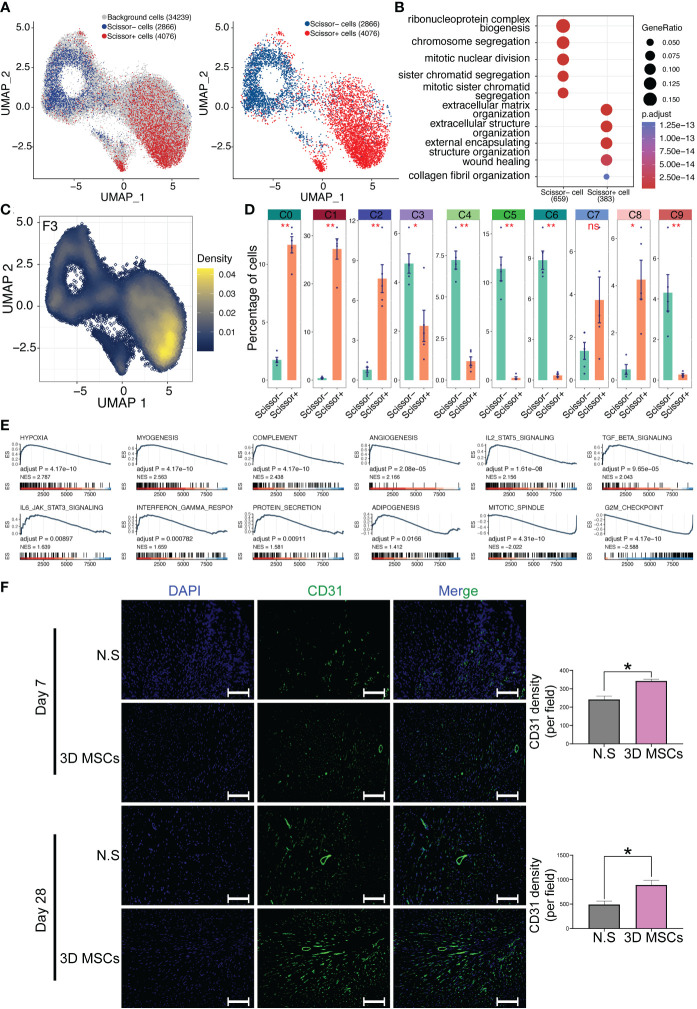
Scissor analysis identifies phenotype-related subpopulation by combing scRNA-seq data and bulk RNA-seq data. **(A)** Scissor analysis identifies a bio-function-related subpopulation: scissor^+^ MSCs, which contained 4076 cells. **(B)** Gene Ontology enrichment analysis for scissor^+^ MSCs and scissor^-^ MSCs. ClusterProfiler package was adopted for GO analysis. **(C)** Expression levels of CD142(F3) visualized by UMAP plot. CD142 was mainly expressed in scissor^+^ MSCs. **(D)** Proportion of scissor^+^ MSCs and scissor^-^ MSCs in all ten clusters. **(E)** GSEA study for scissor^+^ MSCs versus scissor^-^ MSCs. The hallmark database was used and ClusterProfiler package was adopted for GSEA analysis. **(F)** Representative immunofluorescence images of heart tissues. Blue, DAPI; Green, CD31. Scale bar = 100 μm. *p< 0.05, **p< 0.01, ***p< 0.001; n.s., no significant difference.

However, it is still unclear which cells were related to the cardioprotective phenotype even after confirming the heterogeneity of MSCs using canonical single cell analysis pipeline. Hence, the scissor pipeline was leveraged to screening out cardioprotective MSCs (27), which was exclusively developed for linking single cells with particular phenotypes. Successfully, we have identified a scissor^+^ MSC/CD142^+^ MSC subpopulation (4076 cells), a scissor^-^ MSC subpopulation (2866 cells) and other background cells (34239 cells). Of note, the scissor^+^ MSC/CD142^+^ MSC subpopulation (**Figure 4A**) was highly correlated with the cardioprotective phenotype of the high efficacy of cardioprotection of MSCs, which were *in vitro* and *in vivo* validated by Lee et. al (12). More, recently, CD142^+^ MSCs were considered to be capable of repairing tissue and wound healing (14, 39). All these results triggered us to propose that CD142^+^ MSCs might be the major therapeutic subpopulations for post-MI HF. More, enrichment analysis also indicates the wound repairing function of CD142^+^ MSCs (**Figure 4B**). And CD142 was highly expressed in CD142^+^ MSCs (**Figure 4C**). Then we studied the relationship among the identified ten clusters, CD142^-^ MSCs and CD142^+^ MSCs. Interestingly, all ten clusters were significantly different between CD142^-^ MSCs and CD142^+^ MSCs, implying the complexity and heterogeneity of MSCs. Specifically, C0, C1, C2, C7 and C8 were predominantly enriched in CD142^+^ MSCs while C3, C4, C5, C6 and C9 were highly overlapped with CD142^-^ MSCs. Hence, CD142^-^ MSCs and CD142^+^ MSCs were relatively distinct subpopulations and played crucial roles in cardioprotective phenotype of MSCs detected by scissor analysis (**Figure 4D**). More, even though it was not/cannot identified by routine analysis (dimension reduction and clustering analysis using seurat package (25)), it shows greatly relationship and distribution characteristics with the ten clusters. Then we performed GSEA analysis and results showed that CD142^+^ MSCs were related to protein secretions, angiogenesis, myogenesis and hypoxia while CD142^-^ MSCs might be more related to mitotic spindle and G2M Checkpoint (**Figure 4E**), indicating that CD142^+^ MSCs exerted secretory and wound-healing bio-functions while CD142^-^ MSCs took in charge of self-renewal function, collaboratively contributing to cardioprotection in post-MI HF. Then, we detected the expression of CD31, a marker of angiogenesis, and results showed that 3D MSCs could promote the angiogenesis (**Figure 4F**), consistent with the GSEA results of CD142^+^ MSCs and CD142^-^ MSCs.

### 125 combined algorithms for constructing ligand model for identifying secretory potential of MSCs

**Figure 5:**
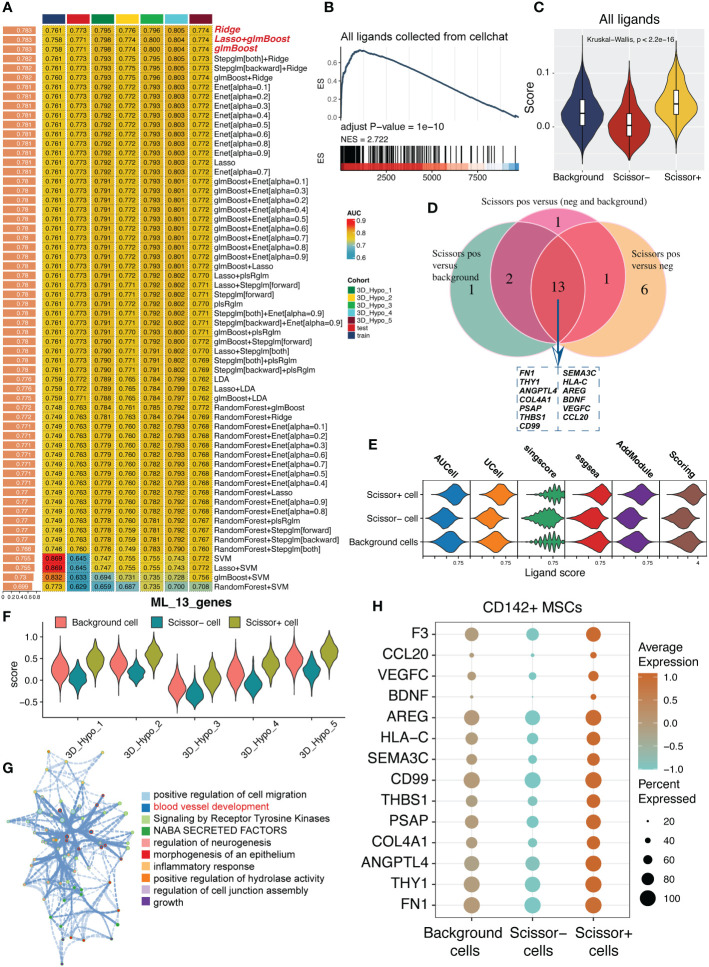
125 combined algorithms for screen out a secretory predictive ligand model for MSCs. **(A)** 125 combined algorithms for identifying a predictive model based on ligands collected from cell chat package. **(B)** GSEA pipeline for confirming the crucial role of ligands in scissor^+^ MSCs. **(C)** Violin plot showing the ligand score calculated using add module function in Seurat package. **(D)** Intersection of feature ligands figured out by using 125 combined algorithms. **(E)** Model score calculated by five algorithms. Scoring is the average values of the five algorithms, containing AUCell, UCell, sing score, ssGSEA and addmodule. **(F)** Violin plot showing the model score value in five samples. Scissor^+^ MSCs expressed higher model score than other MSCs (Scissor^-^ MSCs and background MSCs). **(G)** Function enrichment analysis of the 13 ligands. Metascape server (https://metascape.org) was used to analyze which potential biological activities the 13 ligands participated in. **(H)** Expression levels of the 13 ligands and F3 (CD142) in background MSCs, scissor^+^ MSCs and scissor^-^ MSCs.

After the identification of scissor^+^ MSCs/CD142^+^ MSCs based upon scissor analysis, we tried to develop a predictive model for cardioprotective potential of MSCs. Considering the most of MSCs exerted their therapeutic effects via cell-cell intercommunications (CCI) and depending on ligand-receptor pairs, we applied 125 combined algorithms for screening the most suitable and robust model based on ligands, representing the potential secretome-based therapeutic effect of MSCs (**Figures 5A**, **S3**). To uncover the pivotal role of ligands in scissor^+^ MSCs, we also leveraged gsea algorithm and add module algorithm for verification. As demonstrated in **Figures 5B, C**, all ligands collected from cell chat database were mainly enriched in scissor^+^ MSCs. After comparisons of CD142^+^ MSCs versus background cells, CD142^-^ MSCs and background+CD142^-^ MSCs, respectively (**Supplementary Figure S3**), we then intersected the selected feature ligands and finally 13 ligands were left (**Figure 5D** and **Supplementary Table S1**). Then we calculated the ligand score using five algorithms, results show that scores were consistently up-regulated in CD142^+^ MSCs. And the scores were also consistently up-regulated in CD142^+^ MSCs in five samples, respectively (**Figures 5E, F**). Enrichment analysis using metascape website (https://metascape.org) shows that these ligands were mainly responsible for vascular development, growth and cell migration (**Figure 5G**). Expression levels of these ligands and CD142 were higher in CD142^+^ MSCs (**Figure 5H**). To sum up, the ligand model we developed might be a good tool for estimating or presenting the secretory efficacy of MSCs, also predominantly reflecting the cardioprotection ability of MSCs.

### HdWGCNA pipeline shows the high correlation between the ligand model and MSCs functional module

For better unraveling the ligand model might represent and reflect the therapeutic effects of MSCs, we adopted the hdWGCNA for subsequent analysis. As a result, all genes were classified into seven modules, comprising turquoise, brown, green, red, yellow, blue and black modules (**Supplementary Figure S4**). Intriguingly, scissor^+^ MSCs were mainly related to turquoise, brown and green modules while scissor^+^ MSCs were mainly related to blue module (**Supplementary Figures S5A**, **S5B**). And after calculating the DEGs between scissor^+^ MSCs and scissor^-^ MSCs, the odds ratio also shows the consistent results (**Supplementary Figure S5C**). Of interest, the ligand model based on 13 ligands was also highly related to green, turquoise and brown modules, consistent with scissor^+^ MSCs/CD142^+^ MSCs (**Supplementary Figure S5C**). And the correlation analysis shows that turquoise, brown, red and green modules were highly correlated with each other than other modules (**Supplementary Figure S5D**). Thus, these gene modules might represent main functions of scissor^+^ MSCs/CD142^+^ MSCs. Gene ontology (GO) enrichment analyses of these modules also indicate the related functions, including “extracellular matrix organization”, “regulation of transcription in response to hypoxia”, “endoderm formation” and so on (**Supplementary Figure S5E**). To further prove the phenotype-associated of the ligand model, we performed the correlation analysis and data show that the constructed ligand model was positively correlated with green, brown and turquoise modules, as well as negatively correlated with blue modules (**Supplementary Figure S5F**). Additionally, we also found that VEGFC, COL4A1, THBS1 and FN1 were in turquoise module while CD99, PSAP, ANGPTL4 and THY1 were in green module (**Supplementary Table S2**).

### CD142^+^ MSCs secreted more cardioprotective cytokines and possessed higher cell-cell interactions than CD142^-^ MSCs

**Figure 6:**
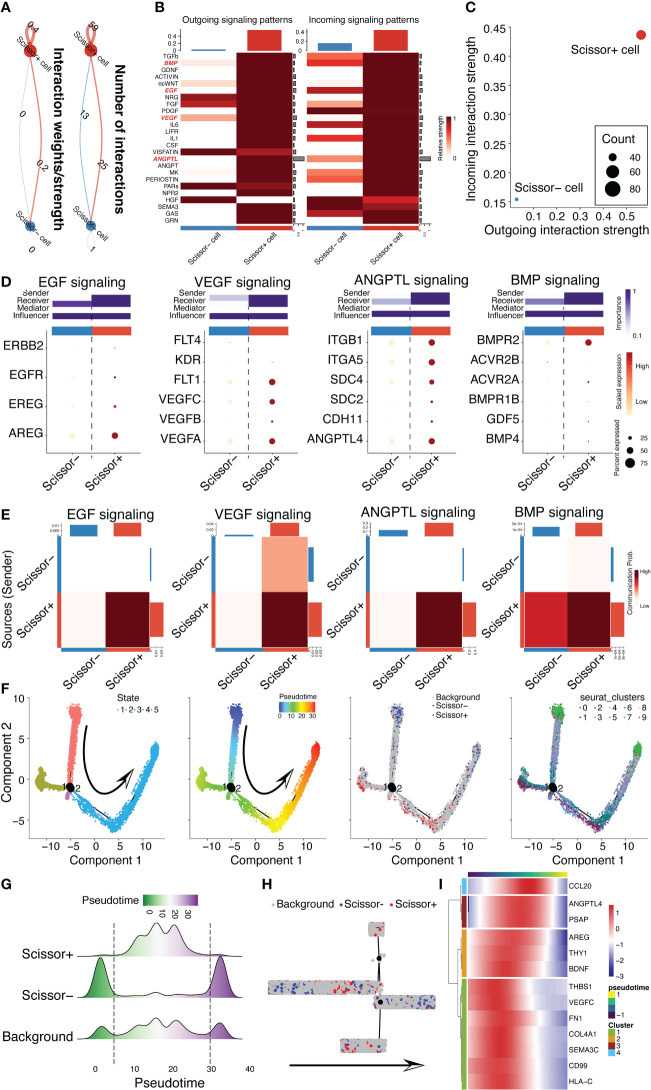
CD142^+^ MSCs/scissor^+^ MSCs were different from scissor^-^ MSCs. **(A)** Cell chat analysis revealed the different cell-cell communication strength of scissor^+^ MSCs and scissor^-^ MSCs. **(B)** Comparison of the outgoing and incoming signaling patterns between scissor^+^ MSCs and scissor^-^ MSCs. BMP, EGF, VEGF and ANGPTL signaling pathways were highlighted in red. **(C)** Scatter plots showed the difference of the outgoing and incoming interaction strengths between scissor^+^ MSCs and scissor^-^ MSCs. **(D)** Dot plot displayed the ligands and receptors in BMP, EGF, VEGF and ANGPTL signaling pathways. The potential roles, comprising sender, receiver, mediator and influencer, were also predicted between scissor^+^ MSCs and scissor^-^ MSCs. **(E)** Communication probabilities between scissor^+^ MSCs and scissor^-^ MSCs. **(F)** Pseudotime analysis for 3D MSCs depended on monocle2 package. The arrow indicates the predicted trajectory of MSCs. **(G)** Ridge plot illustrated the cell distribution of background MSCs, scissor^+^ MSCs and scissor^-^ MSCs following pseudotime inferred by monocle2. **(H)** Distribution of background MSCs, scissor^+^ MSCs and scissor^-^ MSCs in the clustering tree according to pseudotime. **(I)** The expression patterns of 13 ligands following pseudotime.

Cell and cell communications take major parts in the bio-functional characteristics of MSCs, especially in exerting therapeutic effects. Hence, we leveraged cell chat pipeline to analyze the excretive ability of MSC subpopulations. Obviously, we found that CD142^+^ MSCs had stronger interaction power than CD142^-^ MSCs (**Figures 6A, B**), no matter the incoming interaction strength or outgoing interaction strength (**Figure 6C**). Then, we visualized four important signaling pathways related to cardioprotection, including EGF, VEGF, ANGPTL and BMP signalings, which were all high expressed in CD142^+^ MSCs rather than CD142^-^ MSCs (**Figures 6D, E**). Further, pseudotime analysis was conducted to illustrate the relationship between CD142^+^ and CD142^-^ MSCs (**Figure 6F**). Intriguingly, CD142^+^ MSCs were in the early-middle state among the overall pseudotime distribution of all MSCs, indicating that CD142^+^ MSCs were quite different from CD142^-^ MSCs (**Figures 6G, H**). And the 13 ligands following the pseudotime had similarity of the distribution of CD142^+^ MSCs (**Figure 6I**).

### Spatial construction of CD142^+^ and CD142^-^ MSCs using CSOmap algorithm

Now that we have classified the pseudotime distinct distribution of CD142+ and CD142^-^ MSCs, it triggered us to further orchestrate the spatial distribution of CD142^+^ and CD142^-^ MSCs and gain more insights about the difference of the spatial distribution. Therefore, the CSOmap algorithm in MATLAB was utilized for follow analysis. As showed in the organization of 3D space using the calculating three distances to the center, including X, Y, and Z, the CD142^+^ MSCs were closer to the center than CD142^-^ MSCs (**Supplementary Figure S6A**). CD142^-^ MSCs were scattered randomly and distributed stochastically (**Supplementary Figure S6B**). And the density of MSCs also proved the center-distribution character of CD142^+^ MSCs (**Supplementary Figure S6C**). And the distance to center of CD142^+^ MSCs also shorter than CD142^-^ MSCs (**Supplementary Figures S6D**, **S6E**). Connections among CD142^+^ MSCs were much stronger than that among CD142^-^ MSCs (**Supplementary Figures S6F**, **S6G**). Of note, the ligand model we constructed was negatively correlated with the distance to center. In parallel, CD142 was also negatively related to the distance to center (**Supplementary Figure S6H**), demonstrating that the spatial distribution pattern of CD142^+^ MSCs could also be hinted by the optimal ligand model constructed by 125 combined algorithms.

### CD142 and the 13 ligands were both highly related to the functions of MSCs

**Figure 7:**
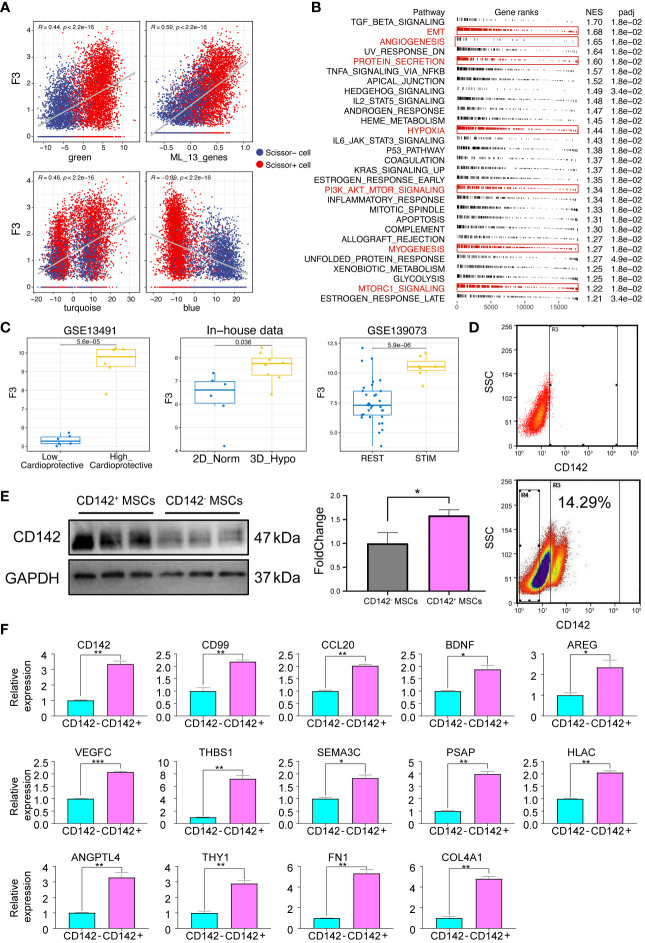
Expression characteristics of CD142 and 13 ligands. **(A)** Correlation analyses between CD142 and green, turquoise, blue module and the ligand model, respectively. Red color represents Scissor^+^ MSCs while blue color indicates Scissor^-^ MSCs. **(B)** GSEA results of CD142. Interested enriched terms were highlighted. **(C)** Expression levels of CD142 (F3) in GSE13491, GSE139073 and our new-sequenced data. **(D)** Flow sorting of CD142^+^ MSCs. **(E)** CD142 protein expression levels detected by Western Blot assay. **(F)** mRNA expression levels of 13 ligands and CD142. * p < 0.05; ** p < 0.01; *** p < 0.001.

Then, we tried to dissect the relationship among CD142 gene (F3), our ligand model and gene modules. Of note, CD142 was positively correlated with green module, turquoise module, ligand model while negatively correlated with blue module (**Figure 7A**). Then, we used the RNA-seq data to perform the GSEA analysis based on the ranking of correlation coefficient with CD142. As shown in **Figure 7B**, CD142 was highly related to angiogenesis, protein secretion, myogenesis and PI3K/AKT-related pathways, which are important for cardiac repair. Then, we also found that CD142 was up-regulated in the High_cardioprotection group from GSE13491 dataset, 3D MSCs group from our sequenced RNA-seq data and stimulated MSCs (pre-conditioned MSCs) from GSE139073, respectively (**Figure 7C**), indicating CD142 might be related to the therapeutic function of MSCs. In parallel, we used flow-sorting to obtain CD142^+^ and CD142^-^ 3D MSCs (**Figure 7D**). Results from Western Blot assay confirmed the high expression of CD142 in CD142^+^ MSCs (**Figure 7E**). Then, we performed qRT-PCR assay to validate the 13 ligands from the established ligand model. Notably, all 13 ligands were up-regulated in CD142^+^ MSCs (**Figure 7F**).

### The ligand model was applicable for robustly estimating secretory power of MSCs in several external cohorts and capable of reflecting the cardioprotective potential of MSCs

**Figure 8:**
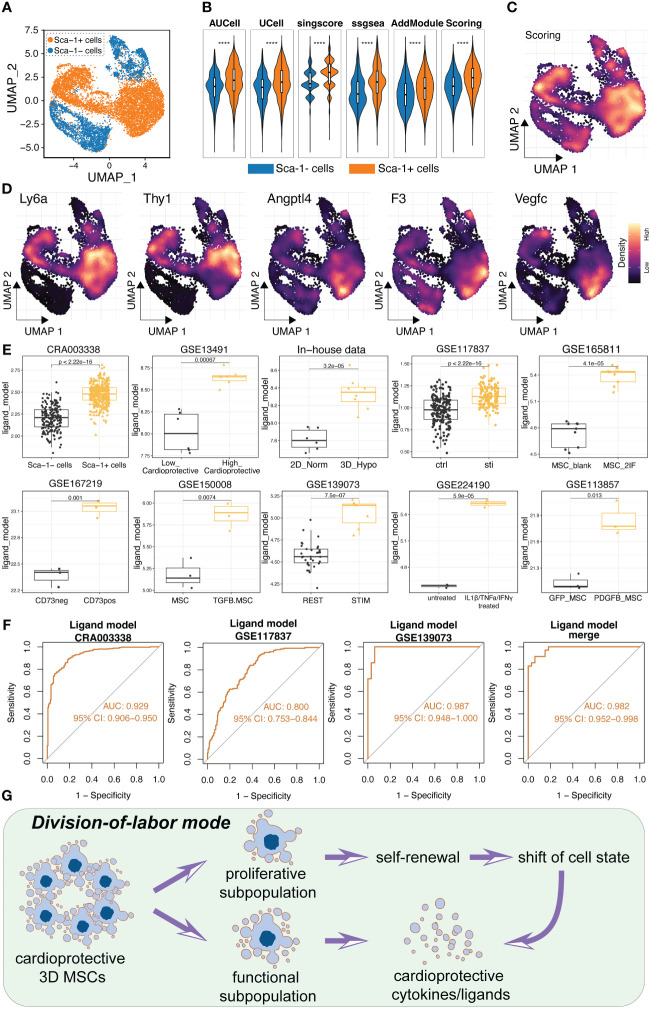
Validation of the ligand model using several datasets, including scRNA-seq, microarray and bulk RNA-seq. **(A)** Single cell analysis of cardiac progenitor cells and two subpopulations were obtained. Sca1^+^ cardiac progenitor cells were *in vivo* and *in vitro* validated as cardioprotective stem cells in previous study. **(B)** Five algorithms were used to calculated the ligand model score between Sca1^+^ and Sca1^-^ cardiac progenitor cells. Scoring value is the average value of the five algorithms. **(C)** Ligand model score was higher expressed in Sca1^+^ cardiac progenitor cells compared to Sca1^-^ cardiac progenitor cells. **(D)** Expression levels of Ly6a (Sca1), Thy1, Angptl4, F3 (CD142) and Vegfc. There genes were all tended to express in Sca1^+^ cardioprotective cardiac progenitor cells. **(E)** Boxplot showed the ligand model score in high-efficacy and low-efficacy MSCs in several public datasets. **(F)** ROC results of the ligand model in several datasets. The small datasets were merged and removed batch effect using sva package. **(G)** Schematic diagram illustrating the potential division-of-labor mode of 3D MSCs. **** p < 0.0001.

Given that we had confirmed the high secretory power of CD142^+^ MSCs and the up-regulation of 13 ligands in CD142^+^ MSCs using flow-sorting and qRT-PCR assays, we want to further confirm the reliability and generalization ability of the ligand model. Hence, several external cohorts were collected for verification. Firstly, our collaborative team had proved that Sca1^+^ cardiosphere-derived cells (CDCs) exert cardioprotective and pro-angiogenic roles while Sca1^-^ cardiosphere-derived cells possess proliferative and angiogenic capabilities (19). These results prompted us to further determine whether the ligand model could also predict the high secretive power and the cardioprotective benefit of Sca1^+^ CDCs. Therefore, we obtained the scRNA-seq data of Sca1^+^ CDCs and Sca1^-^ CDCs (**Figure 8A**). Using several algorithms for scoring the ligand model, we found that the ligand score was stably higher in Sca1^+^ CDCs (**Figures 8B, C**). And CD142, CD90 (Thy1), Angptl4 and Vegfc were also higher expressed in Sca1^+^ CDCs (**Figure 8D**). Additionally, we collected the public open-access RNA-seq or Microarray data related to pre-conditioned MSCs for enhancing the therapeutic and secretory abilities, including GSE117837, GSE165811, GSE167219, GSE150008, GSE139073, GSE224190 and GSE113857 datasets (Detailed information was showed in **Table 1**). Intriguingly, the ligand model score was generally and robustly higher in the function-enhanced MSCs across all ten datasets from our or published datasets (**Figure 8E**). Then, we also calculated the AUC values of ROC in these datasets (datasets with limited samples were merged and removing batch effect) (**Figure 8F**). Results indicated that the ligand model was powerful and useful for estimating the therapeutic and bio-function of MSCs (ALL AUC values > 0.8) (**Figure 8F**). Collectively, the division-of-labor mode of MSCs was illustrated in **Figure 8G**.

### Deployment of a web-server for quickly exploring the transcriptomic characteristics of 3D MSCs and construction of a function-stratification card model for the ligand model

To better visualize our scRNA-seq data of 3D MSCs, we further deployed a web-server using shinycell package (**Supplementary Figure S7**). Readers/researchers could quickly retrieve the expression information of their interested genes in single cell profiling of 3D MSCs (https://wangxc.shinyapps.io/3D_MSCs/). Particularly, to improve the convenience and flexibility of this ligand-based machine learning model in clinical applications, we utilized the Kolmogorov-Smirnov (KS) curve to determine the optimal threshold for evaluating the therapeutic potential of MSCs on the MSC secretory potential assessment card. This card was designed with five distinct grades of secretory power: “Very Low”, “Low”, “Normal”, “High” and “Very High”. Users could only upload the sequenced data and the web-server would quickly predict the therapeutic power of 3D MSCs and output the assessed results (**Supplementary Figure S7**).

## Discussion

Cell therapy is attracting wide attention globally and holds promise as an effective treatment modality for various diseases, including heart failure (40). Compared to adult MSCs, hUC-MSCs are safer, more primitive, exhibit stronger differentiation, proliferation, and immune regulatory abilities, lower immunogenicity, and are conveniently sourced without ethical controversies (9). Therefore, hUC-MSC therapies are increasingly gaining attention from researchers and considered the preferred choice for cell application studies and regenerative medicine.

Recent clinical studies have reported the safety of myocardial transplantation of hUC-MSCs in patients with chronic ischemic cardiomyopathy (41). Our previous study, conducted by our collaborative team, also confirmed the safety of intramyocardial transplantation of hUC-MSCs cultured under routine conditions in mice (16). Furthermore, similar to other teams (42, 43), in this study, we found that clinical-grade 3D MSCs can be generated in high-yield using the 3D and hypoxic culture method. Through experiments *in vivo* and *in vitro*, we have confirmed the significant therapeutic effects of 3D MSCs in treating post-MI HF in mice. However, to date, there remains a lack of definitive supports and reliable findings for standardized and reproducible MSC therapies due to variations in the efficacy of MSC products. Therefore, there is an urgent need for comprehensive and systematic researches on the biomarkers, transcriptomic profiles and functional characteristics of MSCs. Consequently, we conducted scRNA-seq on 3D MSCs to better elucidate their mechanisms of cardiac protection and developed new predictive tools.

The capacity and intensity of interaction between cells secreting bioactive cytokines, and the microenvironment plays a crucial role in tissue homeostasis and wound healing processes. Through screening with 125 combination algorithms, we have identified 13 core key ligand molecules, including FN1, THY1, ANGPTL4, COL4A1, PSAP, THBS1, CD99, SEMA3C, HLAC, AREG, BDNF, VEGFC and CCL20, which were closely associated with cardiac protection and the maintenance of MSCs cellular state. For instances, In zebrafish, a species capable of cardiac regeneration, FN1 derived from the epicardium is necessary for heart repair after injury (44) and epicardial FN1 is highly expressed in ECM, facilitating zebrafish to regenerate cardiac tissues (45). Furthermore, it is widely recognized that FN1 can regulate critical processes, including proliferation, survival and migration, during development and diseases (46, 47). A recent study also reveals that FN1 can promote the maturation of cardiomyocytes in the 3D-engineered heart (48).

THY1 (CD90) is one of the markers for MSCs and has a close relation with the pathogenesis and progression of HF. In the mouse HF model induced by transverse aortic constriction (TAC), the proportion of Thy1^-^ fibroblasts becomes enriched and exhibits a pro-fibrotic phenotype. The mice with *in vivo* knockout of Thy1 display more serious heart dysfunction and fibrosis when suffering HF caused by TAC, suggesting the cardioprotective role of THY1 (49). ANGPTL4 prevents lipoprotein lipase activity (50) and ANGPTL4 overexpression in heart can curb lipoprotein-derived fatty acid delivery (51). On the other hand, Amphiregulin (AREG) produced by Ly6c^low^ cardiac macrophages in cardiac tissue plays a crucial role in compensatory cardiac hypertrophy in a mouse model of pressure overload-induced HF (52). Single-cell sequencing analysis shows that CD8^+^ T cells regulate the conversion of resident and infiltrating macrophages in the heart into cardioprotective macrophages which is pivotal for the myocardial adaptive response. And the cardioprotective macrophages express AREG, which is emphasized in our predictive model (53).

Under stress conditions, BDNF can activate a cascade of signaling pathways, including VEGF, Akt and macrophage activation, exerting a protective effect on ischemic myocardium (54–56). Recombinant BDNF can decrease fibrin fiber density and clot hardness, facilitate clot dissolution (57). VEGF-C is the primary and most effective lymphangiogenic factor and the VEGF-C-VEGFR-3 signaling serves as a crucial regulator of maladaptive hypertrophy, cardiac lymphangiogenesis and HF induced by pressure overload. The therapeutic use of VEGF-C-156S medication has been shown to effectively improve cardiac edema, hypertrophy, and functional impairment (58). Low expression of VEGF-C indicates more severe acute HF and adverse clinical outcomes (59).

In summary, these findings enable us to build a ligand-based predictive model for identifying the potential therapeutic abilities of MSCs. Additionally, we have also discovered the involvement of CD142^+^ MSC subpopulation and the division-of-labor mode of MSCs in cardiac tissue repair during post-MI HF, which might be of great significance for *in vitro* tissue or organ construction, development of novel drug carriers, and clinical treatment of various diseases. Specifically, we identified that 3D MSCs possess a strong secretion capability and exhibit significant heterogeneity and cellular plasticity through scRNA-seq. 3D MSCs were successfully classified into ten clusters and validated by machine learning algorithms. Scissor analysis revealed that scissor^+^/CD142^+^ MSCs may be closely associated with the treatment of heart failure due to their strong secretion of cytokines and higher therapeutic potential, while scissor^-^MSCs/CD142^-^ MSCs primarily contribute to proliferation. Likewise, in our earlier investigation on cardiac progenitor cells, we observed that Sca1^+^ cardiac progenitor cells exhibit the capability to secrete cardioprotective factors, whereas Sca1^-^ cardiac progenitor cells are mainly responsible for proliferation. Despite being different therapeutic cell types, they share remarkably similar cell characters (19). More importantly, scissor^+^ MSCs predominantly express CD142, which is consistent with the literature reporting the wound healing potential of CD142^+^ hUC-MSCs. This might also suggest that CD142^+^ MSCs have the potential to treat post-MI HF through the repair of infarcted tissue (39). Hence, we opted for intramyocardial injection as the mode of treatment for post-MI HF models, instead of intravenous injection. However, the bioinformatics analysis revealed that CD142^+^ MSCs also exhibit robust secretion capacity and CD142^-^ MSCs show the proliferative power, indicating the potential therapeutic effects and cell survival of intravenous MSC administration.

To fully unravel the mechanisms of hUC-MSCs in treating HF, more work needs to be performed. For example, apart from CD142^+^ MSCs, the functions of background cells and CD142^-^ MSCs have not been explored because we have identified ten clusters. Even within the CD142^+^ MSCs, extensive research is needed to uncover their potential cardiac-protective functions by treating the animals with isolated subpopulations of CD142^+^ MSCs to prove their different physiology and clinical usefulness. Furthermore, the interaction between CD142^+^ MSCs and the cardiac microenvironment after injection into the site of cardiac injury has not been experimentally dissected. And for the long term for future cell therapy applications, it is vital to further develop optimal culture conditions to maintain the characteristics of CD142^+^ MSCs, as there are concerns that 3D MSCs may lose their original characteristics under inappropriate *in vitro* culture.

## Conclusion

In summary, our study revealed the cardioprotective potential of 3D MSCs and also found that the division-of-labor mode of 3D MSCs for cardioprotection. Two different subpopulations were in charge of distinct function to make the MSCs survive in cardiac tissue and then ameliorate cardiac fibrosis, enhance heart function and promote angiogenesis. Further, we constructed a ligand-based predictive machine learning model for estimating therapeutic function of MSCs and deployed a web-server for the convenient usage of the model (https://wangxc.shinyapps.io/3D_MSCs/).

## Data availability statement

The data supporting the research findings can be found in the main text and supplementary manuscript. The RNA sequencing experiments are available from the GEO database (https://ftp.ncbi.nlm.nih.gov/geo) and the information was listed in **Table 1**. Interactive web pages for the scRNA-seq data and ligand model are accessible online (https://wangxc.shinyapps.io/3D_MSCs/). The raw data were deposited in the Genome Sequence Archive in National Genomics Data Center, China National Center for Bioinformation/Beijing Institute of Genomics, Chinese Academy of Sciences (BioProject ID: PRJCA022784).

## Ethics statement

The studies involving humans were approved by the Ethics Committee of Shanghai East Hospital of Tongji University. The studies were conducted in accordance with the local legislation and institutional requirements. The participants provided their written informed consent to participate in this study. The animal study was approved by the Ethics Committee of Shanghai East Hospital of Tongji University. The study was conducted in accordance with the local legislation and institutional requirements.

## Author contributions

XW: Writing – original draft, Writing – review & editing. CY: Writing – original draft, Writing – review & editing. XM: Writing – original draft, Writing – review & editing. XL: Writing – original draft, Writing – review & editing. YQ: Data curation, Methodology, Writing – review & editing. ZB: Methodology, Writing – review & editing. YX: Formal analysis, Writing – review & editing. KM: Software, Writing – review & editing. YL: Validation, Writing – review & editing. JS: Investigation, Writing – review & editing. WJ: Funding acquisition, Resources, Supervision, Writing – original draft, Writing – review & editing. ZH: Supervision, Writing – original draft, Writing – review & editing. ZL: Supervision, Writing – original draft, Writing – review & editing.

## Supplementary material

Supplementary Figure 1 Enrichment analyses using KEGG **(A)** and Reactome database **(B)**. Interested enriched terms were highlighted in red color.

Supplementary Figure 2 Enrichment analyses using Gene Ontology database. BP: Biological process; MF: Molecular function; CC: Cellular component.

Supplementary Figure 3 Construction of ligand model using 125 combined algorithms. **(A)** Comparison between scissor^+^ cells and (scissor^-^ and background cells). **(B)** Comparison between scissor^+^ cells and scissor^-^ cells. **(C)** Comparison between scissor^+^ cells and background cells.

Supplementary Figure 4 HdWGCNA pipeline for exploring pivotal gene modules. **(A)** HdWGCNA dendrogram for all detected genes in scRNA-seq data. **(B)** Soft power selection for hdWGCNA. The soft power threshold was set as 4. **(C)** Top genes in each module. **(D)** Gene network for visualizing the relationship among hub genes.

Supplementary Figure 5 High dimensional WGCNA pipeline was leveraged to explore hub functional modules for MSCs. **(A)** Dot plot displaying the module scores identified by hdWGCNA pipeline. There are seven modules identified, containing turquoise, brown, green, red, yellow, blue and black modules. **(B)** Distribution of the interested modules using UMAP plot. **(C)** Odds ratio for screening out crucial modules for scissor^+^ MSCs and scissor^-^ MSCs. And the ligand model was also highly related to green, turquoise and brown modules, similar to scissor^+^ MSCs. **(D)** Correlation analysis among seven modules. **(E)** Gene Ontology enrichment analysis for turquoise, brown, blue and green modules. hdWGCNA: High dimensional weighted gene co-expression network analysis.

Supplementary Figure 6 Reconstruction of the spatial distribution of 3D MSCs. **(A)** 3D spatial characters of 3D MSCs inferred by CSOmap in MATLAB. “X”, “Y” and “Z” values were estimated by CSOmap, representing the cell distances to center in different dimensions. **(B)** 2D spatial distribution of MSCs using “X” and “Y” values. **(C)** Cell density of 3D MSCs following “X” values. **(D)** Different distances to center between scissor^+^ MSCs and scissor^-^ MSCs. **(E)** Different distances to center between scissor^+^ MSCs and scissor^-^ MSCs in five samples. The distances to center of each cell were calculated using “X”, “Y” and “Z” values and the formula was showed in “methods and materials” part. **(F)** Connections between scissor^+^ MSCs and scissor^-^ MSCs. **(G)** Spatial distribution of 3D MSCs using 2D scatter plots. **(H)** Correlation analysis between the distance and ligand model. CSOmap: Cellular Spatial Organization mapper.

Supplementary Figure 7 Deployment of a user-friendly webserver for exploring the scRNA-seq data of 3D MSCs and performing the constructed stratification card model.

Supplementary Table 1 Machine learning identified key ligands and the corresponding receptors collected from cellchat package.

Supplementary Table 2 Relationship between machine learning-identified ligands and hdWGCNA-identified hub genes.

Supplementary Table 3 Primers for all genes used in the qRT-PCR assay.

## Glossary

**Table:**

MI	Myocardial infarction
hUC-MSCs	Human umbilical cord-derived mesenchymal stem/stromal cells
LVFS	Left ventricular fractional shortening
LVEF	Left ventricular ejection fraction
LVEDV/LVESV	Left ventricular end diastolic/systolic volume
LVEDD/LVESD	Left ventricular end diastolic/systolic diameter
COL	Collage
VEGF	Vascular endothelial growth factor
TIMP	Tissue inhibitor of matrix metalloproteinase
MMP	Matrix metalloproteinase
BNP	Brain natriuretic peptide
ANP	Atrial natriuretic peptide
MYH	Myosin heavy chain
THBS1	thrombospondin 1
AREG	Amphiregulin
BDNF	brain-derived neurotrophic factor
ANGPTL4	Angiopoietin like protein 4
KS curve	Kolmogorov-Smirnov curve
COSG	COSine similarity-based marker Gene identification
ISCT	the International Society for Cellular Therapy
Enet	elastic network
Stepglm	stepwise glm
SVM	support vector machine
LDA	linear discriminant analysis
plsRglm	partial least squares linear and generalized linear regression
GBM	generalised boosted regression modelling
NB	Naive_Bayes
hdWGCNA	High dimensional weighted gene co-expression network analysis
CSOmap	Cellular Spatial Organization mapper
BP	Biological process
MF	Molecular function
CC	Cellular component
Sca-1	encoding stem cells antigen-1
CDCs	cardiosphere-derived cells
GMP	Good Manufacturing Practice of Medical Products
Scissor	Single-Cell Identification of Subpopulations with Bulk Sample Phenotype Correlation
H&E staining	Hematoxylin & Eosin staining
N.S	normal saline
