# Supplementary figures and images for: A division-of-labor mode contributes to the cardioprotective potential of mesenchymal stem/stromal cells in heart failure post myocardial infarction
Source: Front Immunol. 2024 Mar 18;15:1363517. doi: 10.3389/fimmu.2024.1363517 (PMC10982400; doi:10.3389/fimmu.2024.1363517)

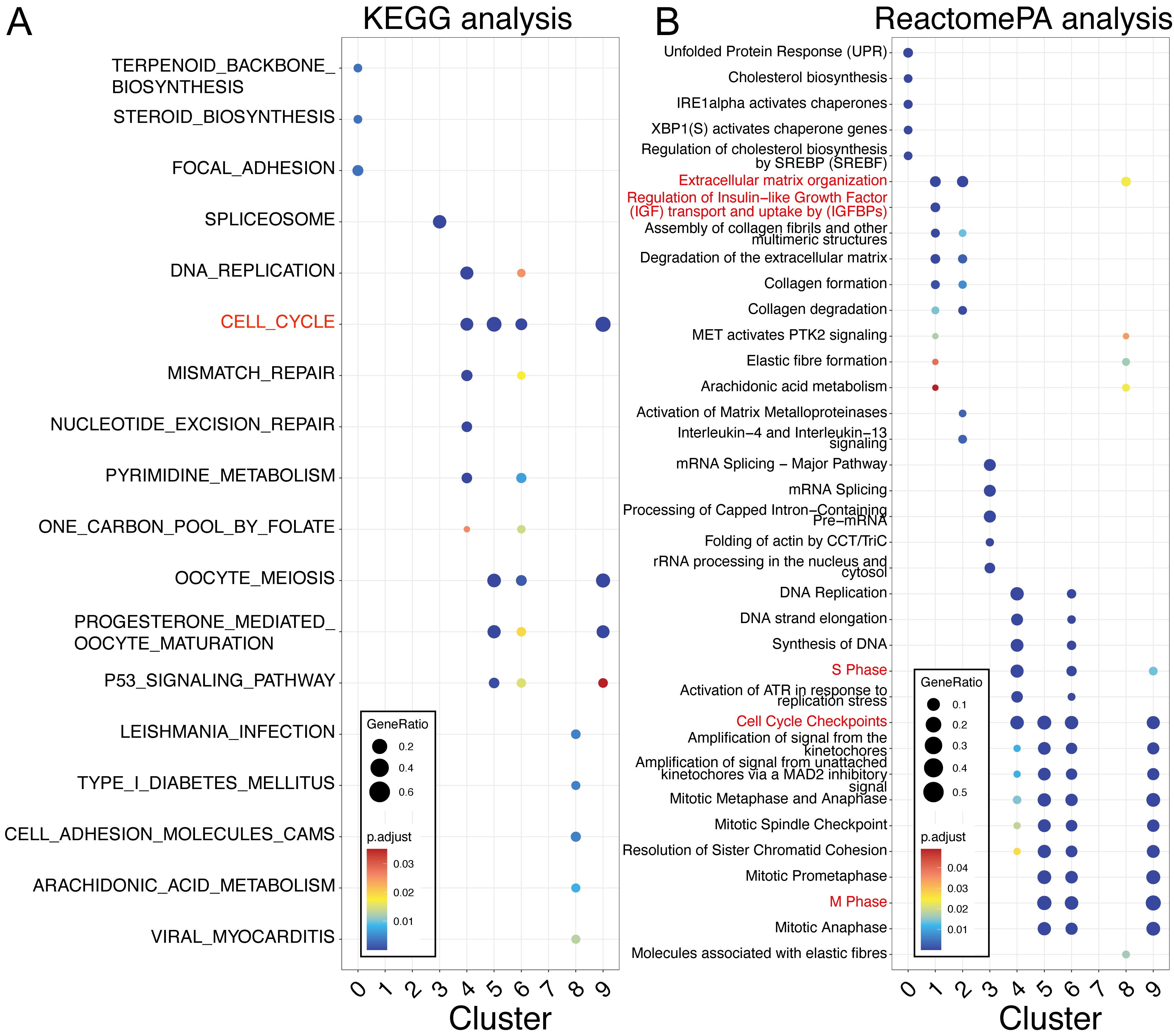

Supplement: Supplementary Figure 1 — Enrichment analyses using KEGG (A) and Reactome database (B). Interested enriched terms were highlighted in red color. [file Image_1.tif]

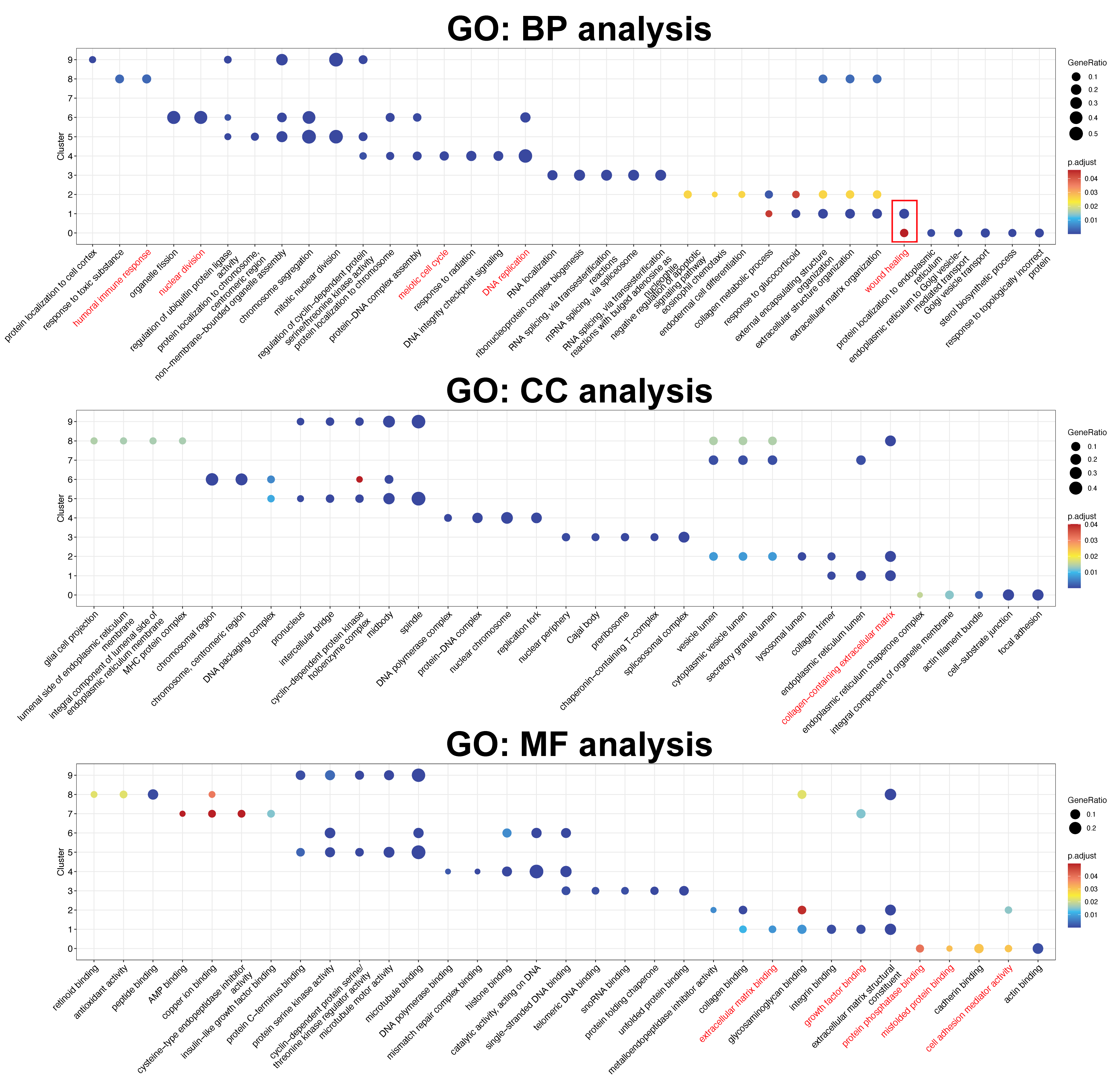

Supplement: Supplementary Figure 2 — Enrichment analyses using Gene Ontology database. BP: Biological process; MF: Molecular function; CC: Cellular component. [file Image_2.tif]

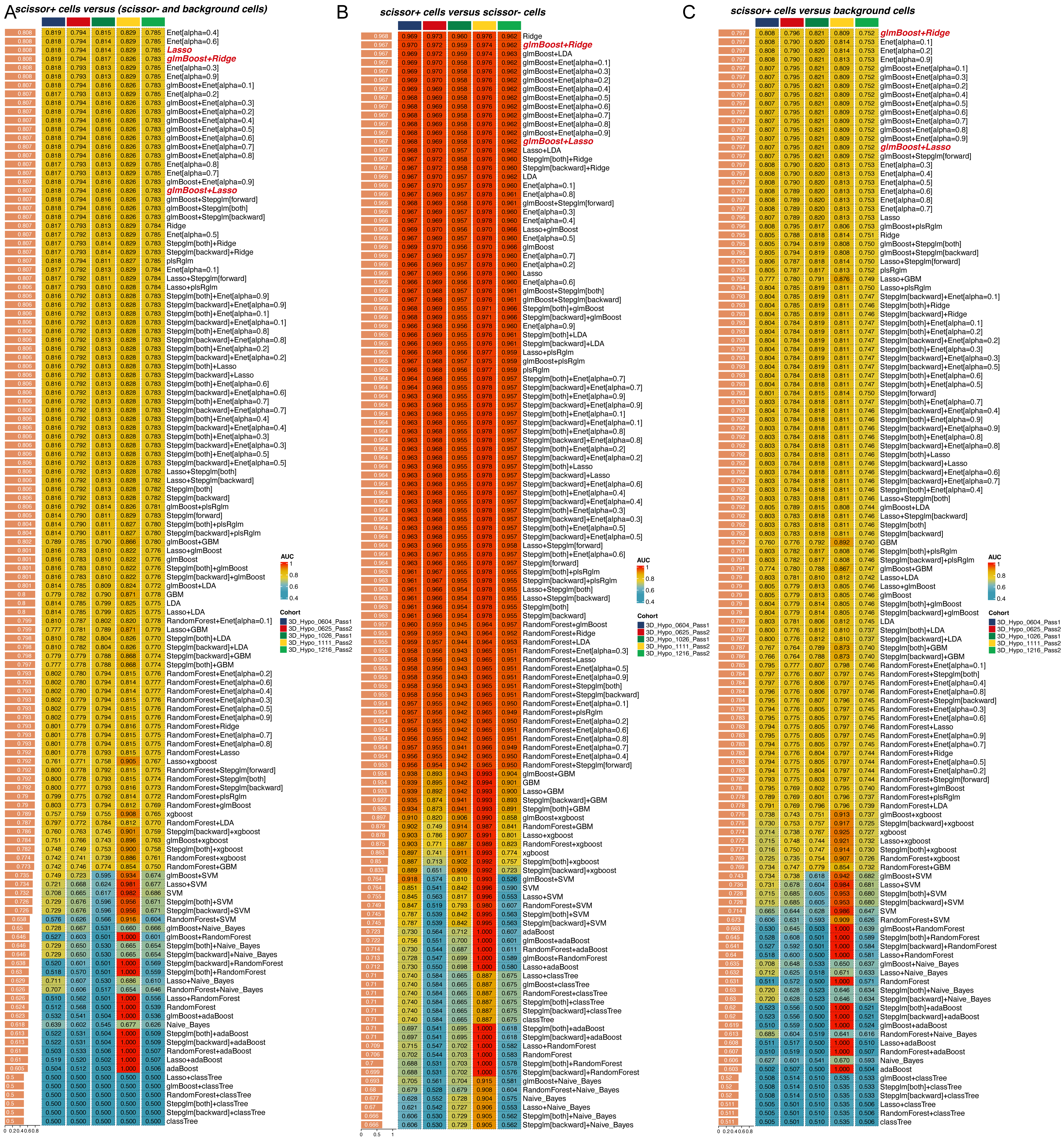

Supplement: Supplementary Figure 3 — Construction of ligand model using 125 combined algorithms. (A) Comparison between scissor+ cells and (scissor- and background cells). (B) Comparison between scissor+ cells and scissor- cells. (C) Comparison between scissor+ cells and background cells. [file Image_3.tif]

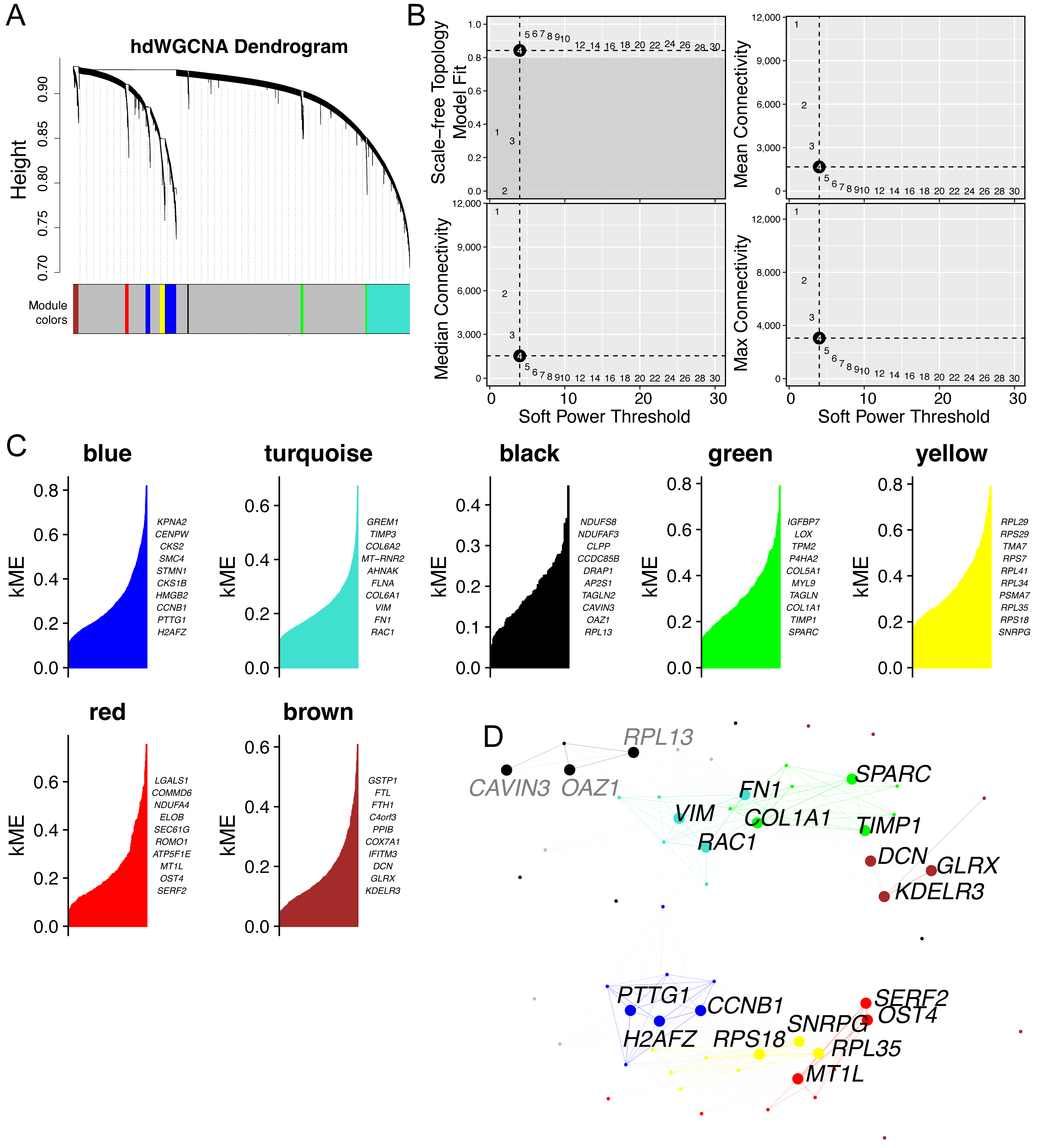

Supplement: Supplementary Figure 4 — HdWGCNA pipeline for exploring pivotal gene modules. (A) HdWGCNA dendrogram for all detected genes in scRNA-seq data. (B) Soft power selection for hdWGCNA. The soft power threshold was set as 4. (C) Top genes in each module. (D) Gene network for visualizing the relationship among hub genes. [file Image_4.tif]

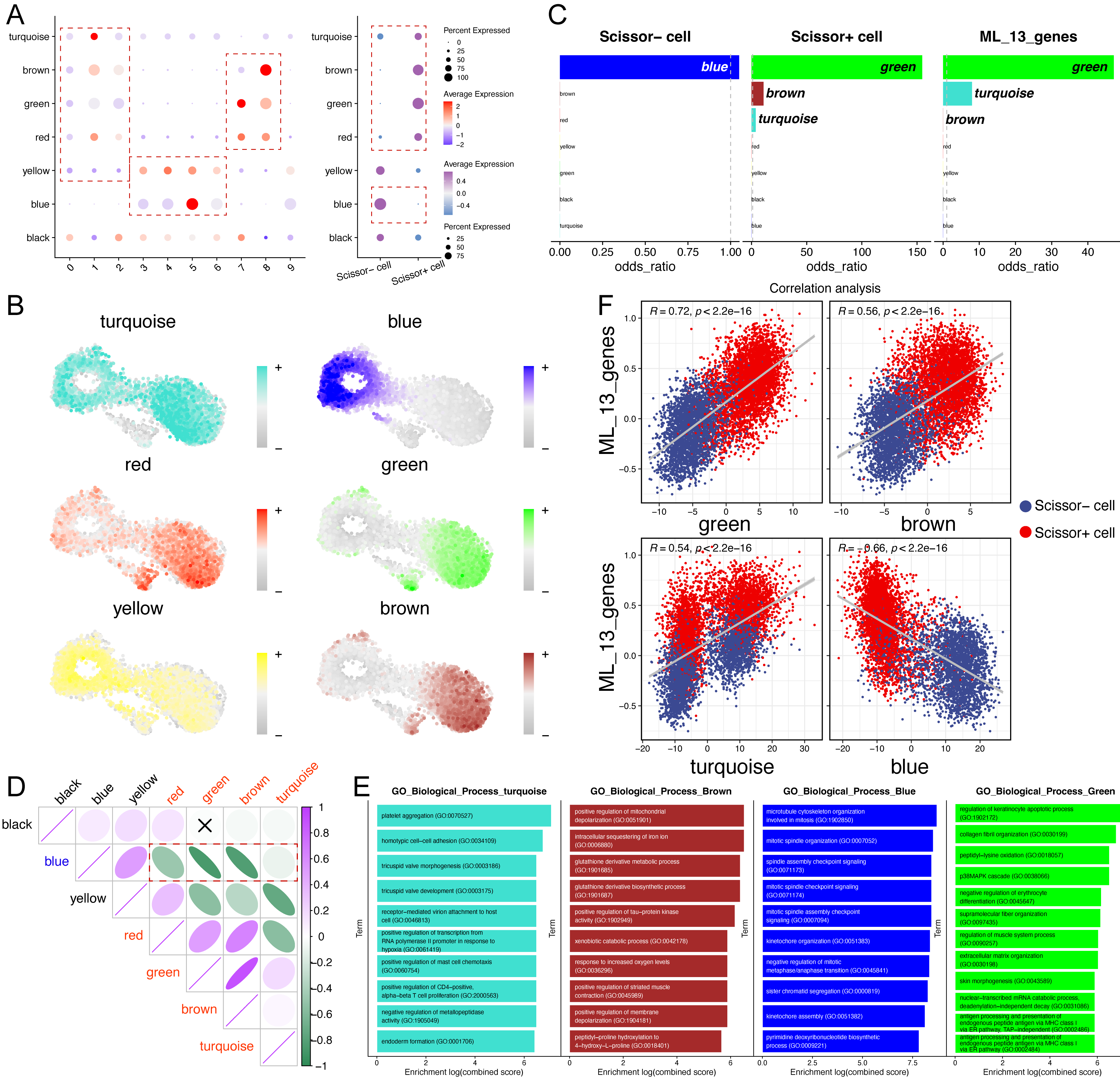

Supplement: Supplementary Figure 5 — High dimensional WGCNA pipeline was leveraged to explore hub functional modules for MSCs. (A) Dot plot displaying the module scores identified by hdWGCNA pipeline. There are seven modules identified, containing turquoise, brown, green, red, yellow, blue and black modules. (B) Distribution of the interested modules using UMAP plot. (C) Odds ratio for screening out crucial modules for scissor+ MSCs and scissor- MSCs. And the ligand model was also highly related to green, turquoise and brown modules, similar to scissor+ MSCs. (D) Correlation analysis among seven modules. (E) Gene Ontology enrichment analysis for turquoise, brown, blue and green modules. hdWGCNA: High dimensional weighted gene co-expression network analysis. [file Image_5.tif]

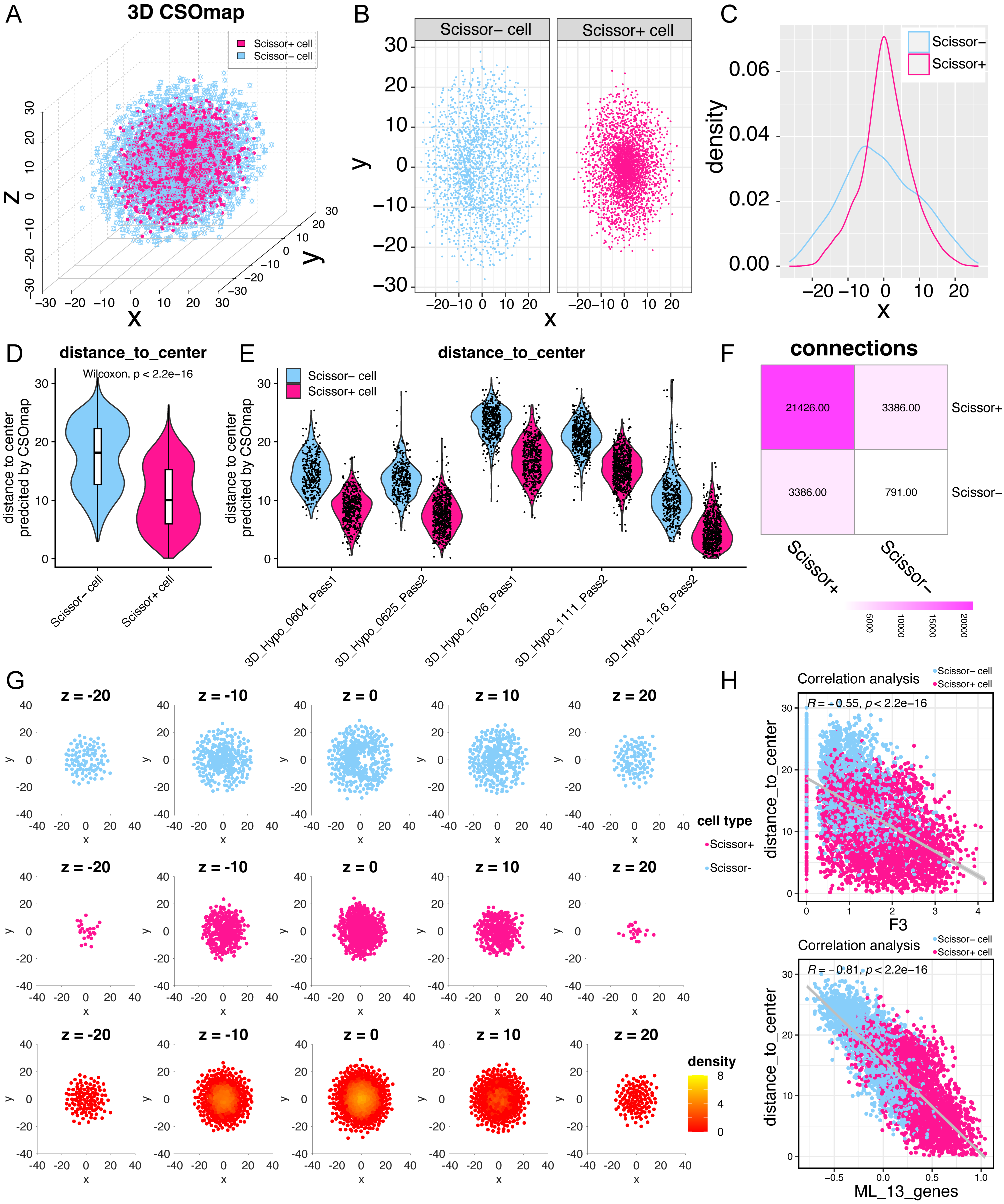

Supplement: Supplementary Figure 6 — Reconstruction of the spatial distribution of 3D MSCs. (A) 3D spatial characters of 3D MSCs inferred by CSOmap in MATLAB. “X”, “Y” and “Z” values were estimated by CSOmap, representing the cell distances to center in different dimensions. (B) 2D spatial distribution of MSCs using “X” and “Y” values. (C) Cell density of 3D MSCs following “X” values. (D) Different distances to center between scissor+ MSCs and scissor- MSCs. (E) Different distances to center between scissor+ MSCs and scissor- MSCs in five samples. The distances to center of each cell were calculated using “X”, “Y” and “Z” values and the formula was showed in “methods and materials” part. (F) Connections between scissor+ MSCs and scissor- MSCs. (G) Spatial distribution of 3D MSCs using 2D scatter plots. (H) Correlation analysis between the distance and ligand model. CSOmap: Cellular Spatial Organization mapper. [file Image_6.tif]

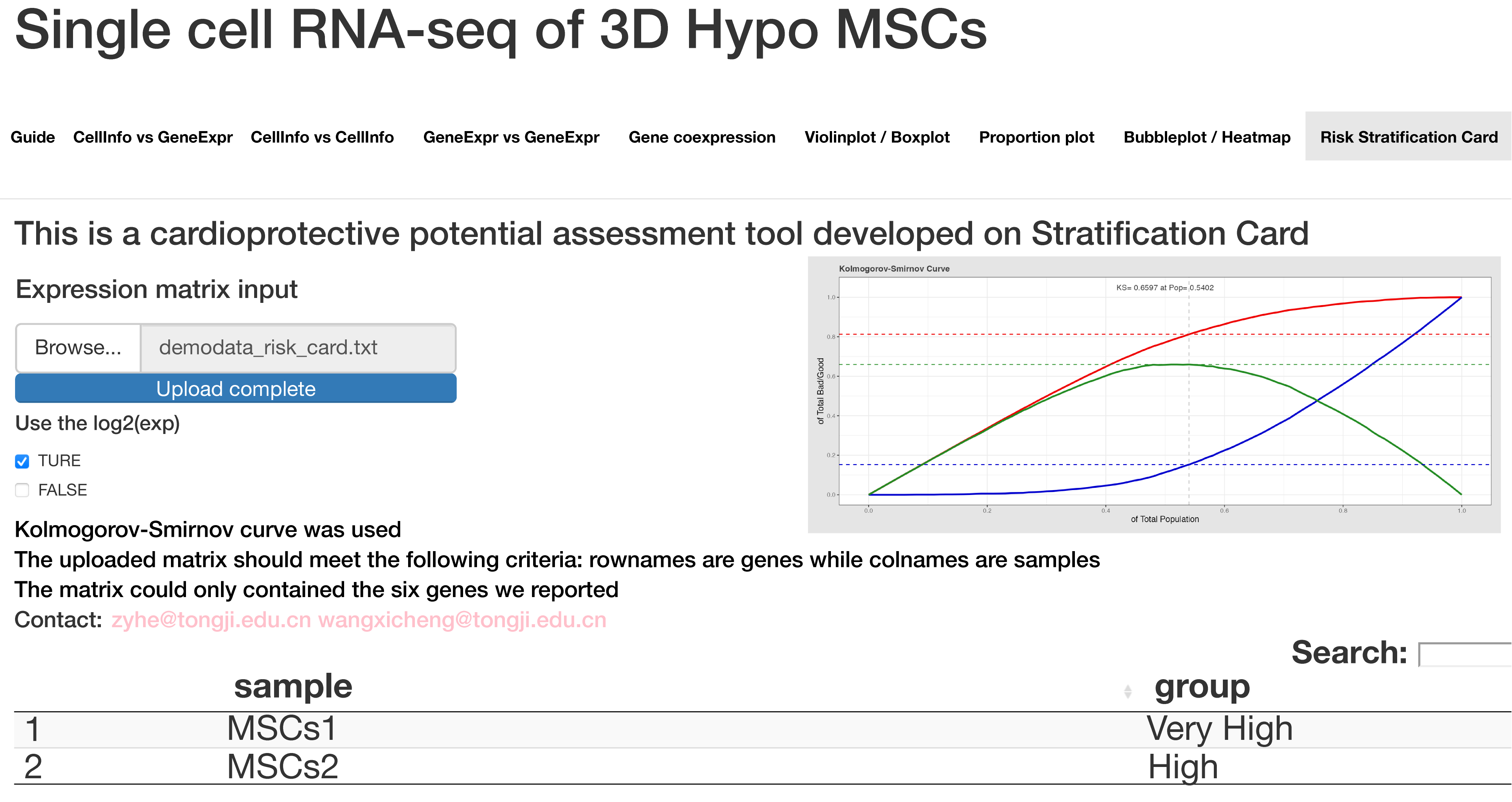

Supplement: Supplementary Figure 7 — Deployment of a user-friendly webserver for exploring the scRNA-seq data of 3D MSCs and performing the constructed stratification card model. [file Image_7.tif]
